# Extracellular Vesicles Mediate Mesenchymal Stromal Cell-Dependent Regulation of B Cell PI3K-AKT Signaling Pathway and Actin Cytoskeleton

**DOI:** 10.3389/fimmu.2019.00446

**Published:** 2019-03-12

**Authors:** Annalisa Adamo, Jessica Brandi, Simone Caligola, Pietro Delfino, Riccardo Bazzoni, Roberta Carusone, Daniela Cecconi, Rosalba Giugno, Marcello Manfredi, Elisa Robotti, Emilio Marengo, Giulio Bassi, Paul Takam Kamga, Giada Dal Collo, Alessandro Gatti, Angela Mercuri, Maddalena Arigoni, Martina Olivero, Raffaele A. Calogero, Mauro Krampera

**Affiliations:** ^1^Stem Cell Research Laboratory, Section of Hematology, Department of Medicine, University of Verona, Verona, Italy; ^2^Proteomics and Mass Spectrometry Laboratory, Department of Biotechnology, University of Verona, Verona, Italy; ^3^Department of Computer Science, University of Verona, Verona, Italy; ^4^Department of Biotechnology, University of Verona, Verona, Italy; ^5^Department of Sciences and Technological Innovation, University of Piemonte Orientale, Alessandria, Italy; ^6^Center for Translational Research on Autoimmune and Allergic Diseases (CAAD), Novara, Italy; ^7^Department of Molecular Biotechnology and Health Sciences, University of Torino, Turin, Italy; ^8^Department of Oncology, University of Torino, Turin, Italy

**Keywords:** extracellular vesicles, mesenchymal stromal cells, B cells, high-throughput analysis, PI3K-AKT signaling pathway, actin cytoskeleton, miRNA-155-5p

## Abstract

Mesenchymal stromal cells (MSCs) are adult, multipotent cells of mesodermal origin representing the progenitors of all stromal tissues. MSCs possess significant and broad immunomodulatory functions affecting both adaptive and innate immune responses once MSCs are primed by the inflammatory microenvironment. Recently, the role of extracellular vesicles (EVs) in mediating the therapeutic effects of MSCs has been recognized. Nevertheless, the molecular mechanisms responsible for the immunomodulatory properties of MSC-derived EVs (MSC-EVs) are still poorly characterized. Therefore, we carried out a molecular characterization of MSC-EV content by high-throughput approaches. We analyzed miRNA and protein expression profile in cellular and vesicular compartments both in normal and inflammatory conditions. We found several proteins and miRNAs involved in immunological processes, such as MOES, LG3BP, PTX3, and S10A6 proteins, miR-155-5p, and miR-497-5p. Different *in silico* approaches were also performed to correlate miRNA and protein expression profile and then to evaluate the putative molecules or pathways involved in immunoregulatory properties mediated by MSC-EVs. PI3K-AKT signaling pathway and the regulation of actin cytoskeleton were identified and functionally validated *in vitro* as key mediators of MSC/B cell communication mediated by MSC-EVs. In conclusion, we identified different molecules and pathways responsible for immunoregulatory properties mediated by MSC-EVs, thus identifying novel therapeutic targets as safer and more useful alternatives to cell or EV-based therapeutic approaches.

## Introduction

Intercellular communication amongst neighboring cells usually occurs either through cell-to-cell contact or exchange of soluble factors. The latter mechanism rarely occurs through the simple secretion of molecules in the intercellular space, which would lead to their rapid inactivation especially if released at tiny concentrations. A very effective, physiological intercellular communication, even at low molecule concentrations, is represented by the exchange of extracellular vesicles (EVs). EVs consist of a membrane-like envelope, i.e., spherical phospholipid bilayer surrounding various molecules, such as proteins, DNA, different types of RNAs (mRNAs, miRNAs, and lncRNAs), lipids, and active metabolites. EVs are shedding vesicles acting as molecular shuttles, constantly released by the cells in a sort of assembly chain process, which are characterized by different size and molecular content according to their origin, biogenesis, and cellular functional status (1–4). EVs include exosomes (50–100 nm), microvesicles (100–1,000 nm), apoptotic bodies (1–5 μm), and some other membrane-bound particles. Exosomes originate from multivesicular body and contain common protein families, such as chaperones (Hsp70 and Hsp90), cytoskeletal proteins (actin, myosin and tubulin), ESCRT proteins (TSG-101 and Alix), proteins involved in transport and fusion (Rab11, Rab7, Rab2, and Annexines) as well as tetraspanin proteins (CD9, CD63, CD81, and CD82) (5–7). Microvesicles result from plasmatic membrane gemmation and contain specific cytoplasmatic proteins of the cells of origin, such as GTP-binding protein, ADP-ribosylation factor 6 (ARF6), matrix metalloproteinases (MMPs), glycoproteins (e.g., GPIb, GPIIb-IIIa), integrins, receptors (e.g., EGFRvIII), and cytoskeletal components (e.g., β-actin and α-actinin-4) (5, 6). In addition, both exosomes and microvesicles contain a large number of molecules, whose functions are still under investigation (8). By contrast, apoptotic bodies are functionally different by the other two kinds of EVs, as they result from the programmed cell death mechanisms and contain DNA, histones, and cellular debris derived from cell dismantlement; their formation is a highly controlled mechanism preventing leakage of potentially toxic, enzymatically active or immunogenic components of dying cells, thereby preventing tissue destruction, inflammation, and autoimmune reactions through cytoskeleton weakening and activation of caspase enzymes. In addition, apoptotic bodies act as a powerful signaling pathway for the microenvironment surrounding dying cells (1–4).

The secretion of EVs is not restricted to mammalian cells, but it has been also identified in lower eukaryotes and prokaryotes, highlighting a high degree of conservation of such communication system and, consequently, its relevance in intercellular communication (9, 10). EVs influence various biological process directly activating cell surface receptors through protein and bioactive lipid ligands, and delivering effectors including transcription factors, oncogenes, mRNAs, and non-coding regulatory RNAs, such as miRNAs into target cells (11–13).

EVs play a role also during the onset of immune responses. EVs possess immunosuppressive effects on T cells and NK cells and play a crucial role in the induction of regulatory T and myeloid cells to further inhibit the immune response (14–16). EVs in co-culture with peripheral blood mononuclear cells (PBMCs) inhibit B cell proliferation and immunoglobulin release (17). Placenta-derived exosomes, purified from the blood of pregnant women, carry immunosuppressive molecules, such as Fas-ligand, which induce tolerance toward the fetus (1–4). On the other hand, EVs may also stimulate the immune system, with the final effect depending on many factors, such as effector and target cells as well as the biological context in which this interaction takes place (1, 4, 18).

Even the immune regulatory properties of stromal cells can be influenced by EV release, as shown by a number of data obtained with mesenchymal stromal cells (MSCs) derived from bone marrow (BM) or other tissues (19, 20). MSCs are adult, multipotent cells of mesodermal origin representing the progenitors of all stromal tissues. In fact, although they were first identified and isolated from BM, MSCs can be expanded from virtually any tissue with a stromal architecture, including adipose tissue, umbilical cord blood, skin, tendon, muscle, and dental pulp (21–24). MSCs are characterized by the ability to differentiate into mesodermal tissues, such as osteoblasts, chondrocytes, and adipocytes, with therapeutic potential in the field of regenerative medicine (25, 26). However, MSCs possess also significant and broad immune modulatory functions affecting both adaptive and innate immune responses once MSCs are primed by the inflammatory microenvironment (27). MSC-mediated immune regulation has been confirmed by both preclinical and clinical studies based on systemic or local MSC administration in a broad spectrum of inflammatory and autoimmune diseases, such as Graft-vs.-Host Disease (GvHD) (28), Crohn's disease and enteropathies (29–31), as well as in cardiovascular disease (32, 33), acute kidney injury (34), colitis, sepsis (35), and other disorders (36). The beneficial effect of MSCs was initially ascribed to their ability to home within the inflammation sites, thus stimulating endogenous repair of the injured tissue and modulating immune responses (37–39); actually, only a negligible percentage of systemically administered MSCs is capable of reaching the damaged tissues (40, 41). Therefore, MSC biological activity–in terms of both regeneration and immunomodulation–is supposed to reside in their ability to act at paracrine level through the release of bioactive factors, as observed with cardiomyocytes exposed to hypoxia/reoxygenation (42), or through indirect mechanisms involving phagocytes (43). Recently, the role of EVs in mediating the paracrine effects of MSCs has been recognized both in tissue repair and immunomodulation (19, 20). Nevertheless, the molecular mechanisms responsible for the immunomodulatory role of MSC-derived EVs are still poorly characterized. Therefore, in this study we carried out a high-throughput characterization of miRNA and protein expression in MSCs at resting conditions (control MSCs, cMSCs), inflammatory conditions (primed-MSCs, pMSCs), and corresponding EVs (cEVs and pEVs, respectively). We then functionally validated the modulated molecules and we focused our analysis on those involved in the crosstalk between MSCs and B cells for the following reasons: (i) we and other groups have previously demonstrated that B cell activity is strongly modulated by EVs released by MSCs (20, 44); (ii) the characterization of the EV-mediated interactions between normal B cells and MSCs represents the basis for studying the same crosstalk in inflammatory and autoimmune diseases as well as in hematological malignancies (45–47). The molecules and pathways here identified are important for the immunoregulatory properties mediated by MSC-EVs and may represent some novel potential therapeutic targets as safer and more useful alternatives to cell or EV-based therapeutic approaches for inflammatory, autoimmune and proliferative disorders.

## Results

### Size and Surface Marker Characterization of MSC-Derived EVs

**Figure 1:**
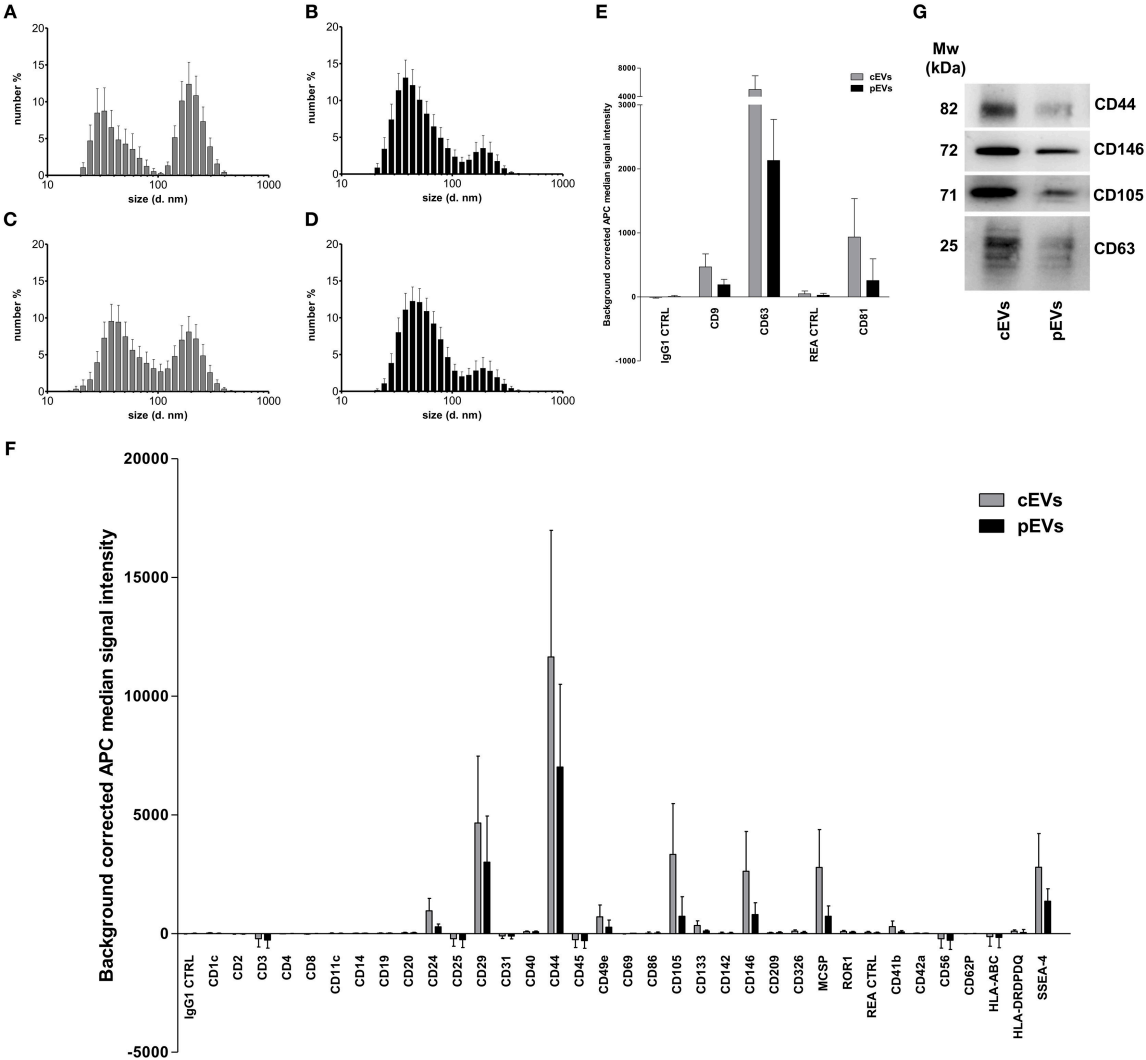
Size and surface marker characterization of MSC-derived EVs. Histograms represent hydrodynamic diameter distribution plots measured on EVs freshly isolated from resting **(A)** and primed MSC **(B)** (cEVs exosomes peak 40.43 ± 15.63 nm, percentage 46.6%; cEVs microvesicles peak 207.7 ± 53.95 nm, percentage 53.4%; pEVs exosomes peak 51.17 ± 23.23 nm, percentage 75.1%; pEVs microvesicles peak 200.9 ± 63.57 nm, percentage 24.9%). **(C)** Hydrodynamic diameter distribution of cEV stored at −80°C (cEVs exosomes peak 51.08 ± 21.07 nm, percentage 59%; cEVs microvesicles peak 196.9 ± 65.34 nm, percentage 41%). **(D)** Hydrodynamic diameter distribution of pEV stored at −80°C (pEVs exosomes peak 55.91 ± 21.99 nm, percentage 83.6%; pEVs microvesicles peak 201.1 ± 76.45 nm, percentage 16.4%). Error bars represent mean ± SD obtained from at least five measurements of three independent samples. All experiments were performed in PBS at 25°C. **(E)** Background corrected median fluorescence intensity of CD9, CD63, CD81 markers and corresponding isotype controls on cEVs and pEVs (*n* = 5). **(F)** Background corrected median fluorescence intensity of 34 surface epitopes on cEVs and pEVs (*n* = 5). **(G)** Immunoblot analysis of CD44, CD146, CD105, and CD63 expression in cEVs and pEVs. This blot is representative of three independent experiments showing the same trends.

**Figure 2:**
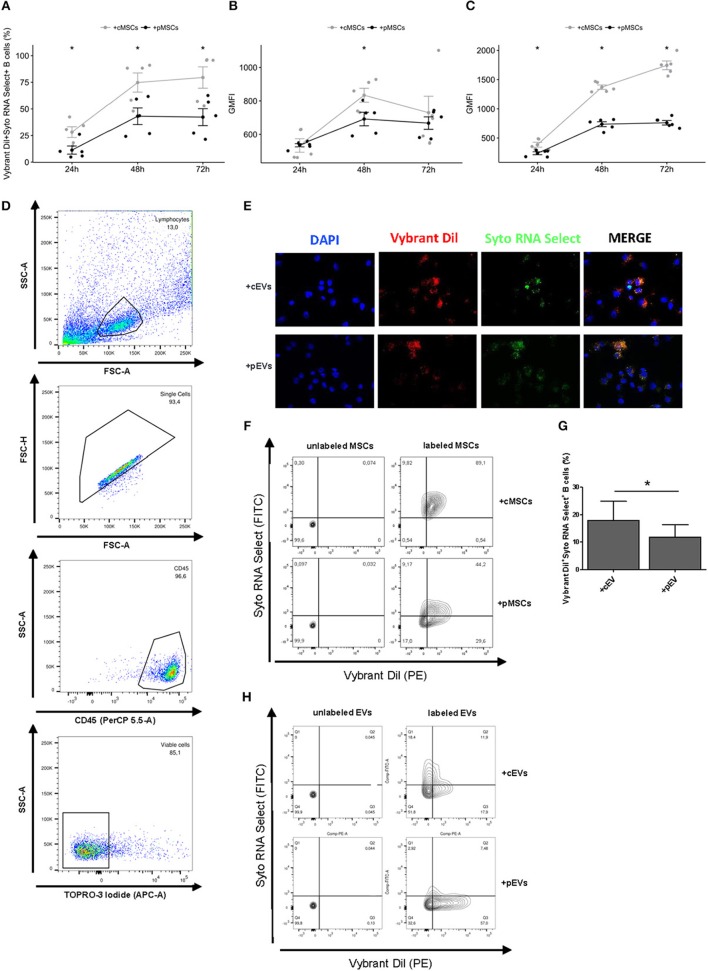
Incorporation of MSC-EVs and RNA transfer in activated B lymphocytes. **(A)** Percentage of Vibrant DiI^+^ Syto RNA Select^+^ B cells co-cultured for 24, 48, and 72 h with double stained resting or primed MSCs (*n* = 5) *p < 0.05. **(B)** Vybrant Dil Geometric Mean of Fluorescence Intensity (GMFI) of B cells co-cultured with double stained resting or primed MSCs. **(C)** Syto RNA Select GMFI of B cells co-cultured with double stained resting or primed MSCs. **(D)** Representative gating strategy on the final gated population. **(E)** MSC-EVs were double-stained for membrane in red (Vybrant Dil) and for RNA in green (Syto RNA Select). Labeled EVs were incubated for 24 h on activated B lymphocytes. The four panels show (from the left to the right) B cells stained with DAPI (blue), the internalization of membrane components of cEVs and pEVs (red), the distribution of Syto RNA Select carried by MSC-EVs inside B cells (green), and a merge between the three previous panels (original magnification 400x). The images are representative for three independent experiments with similar results. **(F)** Representative FACS analysis of Vibrant DiI^+^ Syto RNA Select^+^ B cells co-cultured with double stained (right) or not (left) resting or primed MSCs. **(G)** Percentage of Vibrant DiI^+^ Syto RNA Select^+^ B cells co-cultured for 24 h with double stained resting or primed MSC-EVs (*n* = 5) *p < 0.05. **(H)** Representative FACS analysis of Vibrant DiI^+^ Syto RNA Select^+^ B cells co-cultured with double stained (right) or not (left) resting or primed MSC-EVs.

To characterize MSC-derived EVs, we first analyzed their size using dynamic light scattering (DLS). We identified two separate, equally represented populations of EVs derived from resting MSCs (Figure 1A). The small-sized EVs, ranging from 30 to 100 nm (peak 40.43 ± 15.63 nm, percentage 46.6%), corresponded to exosomes. The large-sized population of EVs, ranging from 100 to 400 nm (peak 207.7 ± 53.95 nm, percentage 53.4%), corresponded to microvesicles. The percentage of exosomes was significantly higher as compared to microvesicles in EVs derived from primed MSCs (75.1 and 24.9%, respectively) (Figure 2B). Furthermore, we re-analyzed the size of EVs after freezing and thawing the samples. We observed the same DLS profile, demonstrating that EVs maintained their integrity (Figures 1C,D). To assess the surface marker profile of MSC-derived EVs, we used a multiplex bead-based platform that allows the detection of 37 surface epitopes. Both resting and primed-EVs were positive for the well-established exosome markers CD63, CD81, and CD9 (Figure 1E). In particular, CD63 intensity was higher compared to CD81 and CD9 expression. As the cells of origin, MSC-derived EVs were positive for CD29, CD44, CD105, and negative for hematopoietic, epithelial, and cancer stem cell markers (Figure 1F). Moreover, MSC-derived EVs expressed SSEA-4 (Figure 1F), an early embryonic glycolipid antigen identifying the adult MSC population derived from BM (48). Furthermore, we found a significant expression of CD146 (Figure 1F), a typical molecule characterizing a specific subpopulation of MSCs with a higher therapeutic potential (49). Western blot analysis confirmed the surface marker profile of EVs, as far as the exosome marker CD63 and the hMSC-associated markers CD44, CD105, and CD146 are concerned (Figure 1G).

### MSC-Derived EV Internalization by Activated B Lymphocytes

To evaluate a possible role of MSC-derived EVs in modulating B cell activity, we first assessed the potential of MSCs to transfer membrane fragments and RNA molecules to activated B lymphocytes. To this aim, activated B lymphocytes were co-cultured with resting or primed MSCs labeled or not at membrane (Vibrant DiI) and RNA (Syto RNA Select) level with fluorescent probes. The transfer of MSC-derived membrane and RNA was observed at different culture times by flow cytometry. We detected a double-positive B cell population receiving MSC-derived EVs containing RNA (Figures 2D,F). EVs derived from both resting and primed MSCs were internalized by activated B lymphocytes. Initial incorporation was observed after 24 h of co-culture, followed by an increase until 72 h. At each time points we observed a higher internalization of cEVs compared to pEVs (Figure 2A). The same trend was observed considering separately the internalization of MSC-derived membranes and RNA molecules, with a more marked effect on RNA transfer (Figures 2B,C). Considering that pMSCs release a higher percentage of exosomes compared to cMSCs, which represent smaller sized-EVs compared to microvesicles, the difference observed in terms of EV internalization may result from the difference in size of internalized particles. To further confirm our hypothesis, we directly co-cultured activated B cells with double-labeled resting or primed EVs and the same experiments were carried out by flow cytometry. As expected, after 24 h we observed a higher internalization of cEVs compared to pEVs (Figures 2G,H). EV incorporation was also assessed by fluorescence microscopy (Figure 2E). Overall, our data showed that the uptake of MSC-derived EVs occurs at both resting and primed conditions, thus highlighting a possible involvement of EVs in modulating B cell activity.

### Proteomic Profiling of MSCs and EVs

To identify proteins potentially involved in immunomodulatory properties mediated by pEVs, MSCs treated or not with inflammatory stimuli and corresponding EVs were analyzed using shotgun MS. A complete list of the identified proteins is shown in Table S1. As expected, the number of identified proteins in the cells is higher compared to that identified in the EV compartment. Nevertheless, the number of modulated proteins is similar among the two compartments (Figure S1 and Table S2).

**Figure 3:**
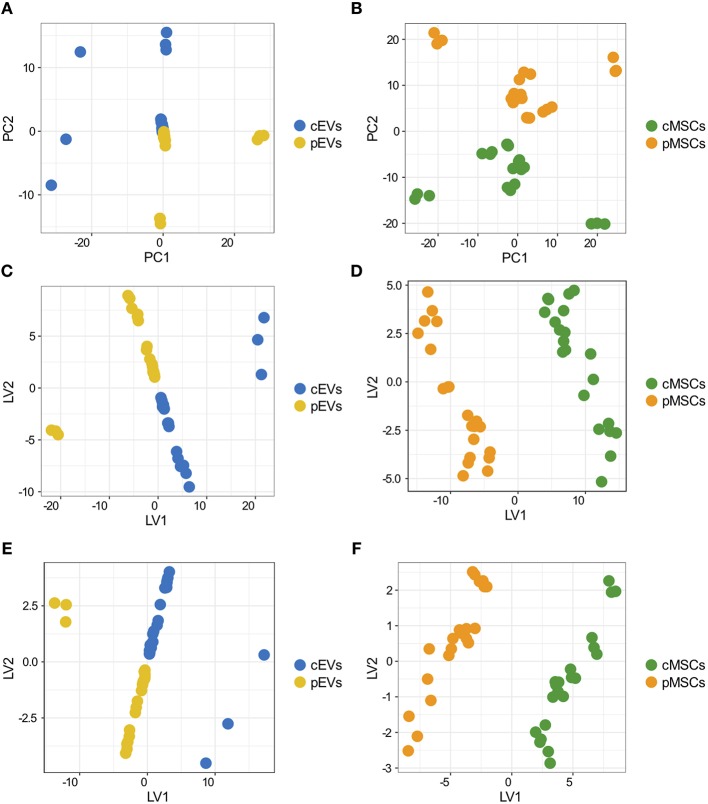
Overall variation of differentially expresses proteins in MSCs and EVs following inflammatory priming and PLS-DA. Score plot of the first two PCs calculated after the application of PCA to EVs **(A)** and MSCs **(B)**. Score plot of the first two LVs calculated after the application of PLS-DA to the 181 proteins selected by Ranking-PCA on the EVs dataset **(C)** and on the first 200 proteins selected by Ranking-PCA on the MSCs dataset **(D)**. Score plot of the first two LVs calculated after the application of PLS-DA to the 55 proteins selected by univariate statistics on the EVs dataset **(E)** and on the 39 proteins selected by univariate statistics on the MSCs dataset **(F)**.

To assess the overall variation of the samples following inflammatory stimuli, shotgun MS data were processed by PCA. The score plot of the first two PCs calculated for EVs (Figure 3A) and MSCs (Figure 3B) allows the clear separation of the sample groups, thus indicating that the information about the inflammatory stimuli is present in the protein profile of both EVs and MSCs samples; nevertheless, in the case of EVs, two samples (BM004 and D24) seem quite different as they are far from the others located at the origin of the axes.

We analyzed differentially expressed proteins in pEVs and pMSCs using both univariate and multivariate approaches. By using the univariate analysis, we found 55 and 39 proteins differentially expressed in pEVs and in pMSCs, respectively (Table S3). Using the multivariate approach, based on Ranking-PCA followed by PLS-DA, we found 181 and 363 proteins differentially expressed in pEVs and in pMSCs, respectively, considering 1 PC in Ranking-PCA and 1 LV in the final PLS-DA models (for MSCs, only the first 200 proteins selected by Ranking-PCA were included in the PLS-DA model). Figures 3C,D report the score plots of the first 2 LVs for EVs and MSCs, respectively. Selected proteins clearly separate resting from primed samples (BM0004 and D24 EVs samples are no more separated from the others as in PCA analysis). The obtained models allowed the perfect classification of all the replications of all the samples (NER% = 100%, accuracy = 100%), both in fitting and in cross-validation for both EVs and MSCs. The list of candidate biomarkers identified by multivariate analysis and the corresponding regression coefficients are provided in Table S4. Variables with a positive coefficient correspond to up-regulated proteins, while those characterized by a negative coefficient correspond to down-regulated proteins. The variables can be ordered according to a decreasing discrimination ability on the basis of the decreasing absolute value of the regression coefficient. The multivariate models were compared to PLS-DA models built including the markers identified as statistically significant by classical univariate statistics (Figures 3E,F) and provided the same classification performances (100% in fitting and cross-validation for all the samples).

**Figure 4:**
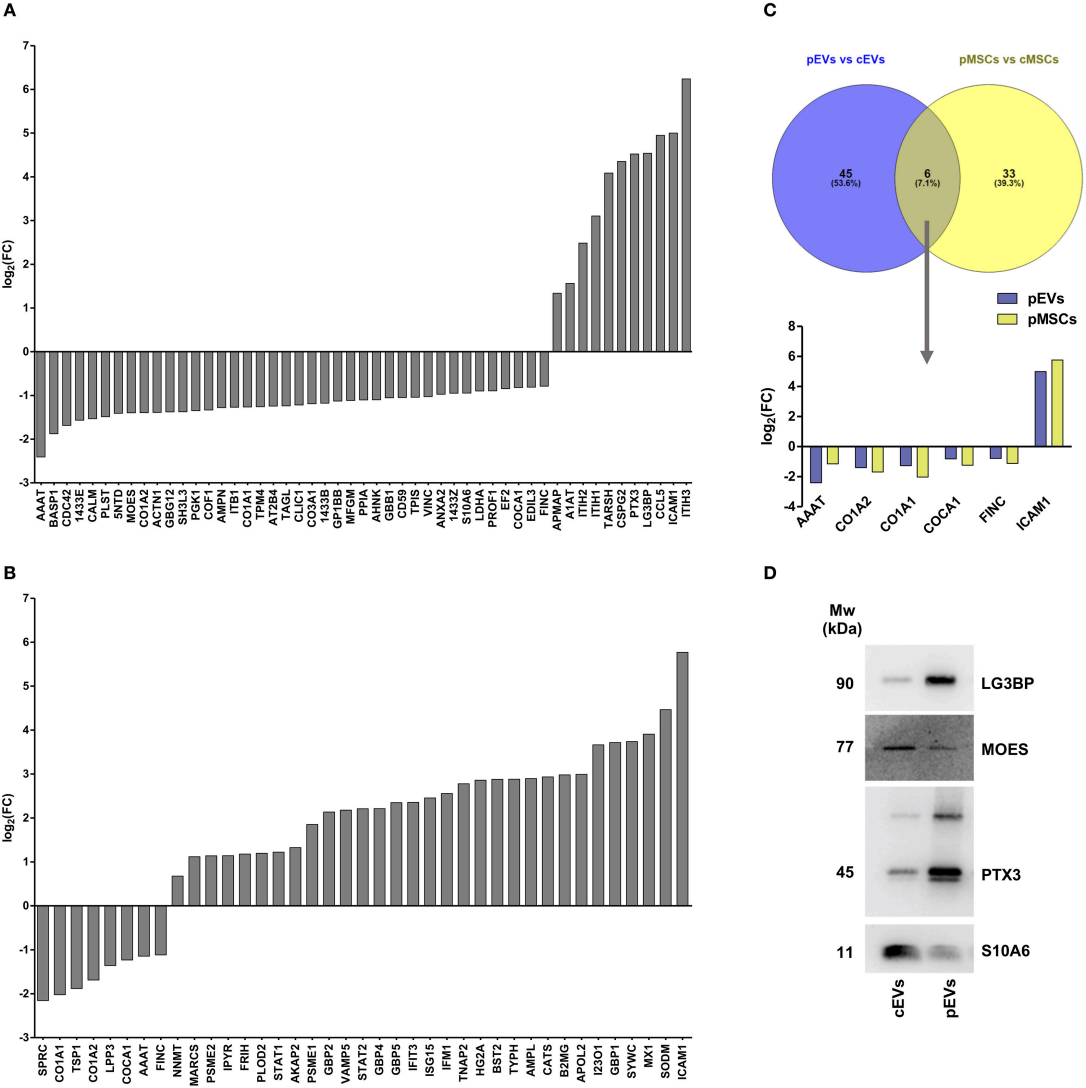
Proteomic profile of resting and primed MSCs and corresponding EVs. **(A)** Differentially expressed proteins in pEVs compared to cEVs obtained both from univariate and multivariate approach (adj *p* < 0.05, Fold Change (FC) > 1.5 and FC < 0.667) (*n* = 7). **(B)** Differentially expressed proteins in pMSCs compared to cMSCs obtained both from univariate and multivariate approach (adj *p* < 0.05, FC > 1.5, and FC < 0.667) (*n* = 7). **(C)** Venn diagram representing modulated proteins both in pMSCs and pEVs. **(D)** Immunoblot analysis of LG3BP, MOES, PTX3, and S10A6 expression in cEVs and pEVs. This blot is representative of three independent experiments showing the same trends.

For downstream analyses, we selected significantly modulated proteins obtained using both univariate and multivariate approaches: thus, we found 51 (Figure 4A) and 39 (Figure 4B) modulated proteins in pEVs and pMSC respectively. Notably, AAAT, CO1A1, CO1A2, COCA1, FINC, and ICAM1 were the 6 proteins modulated both in pMSCs and pEVs showing the same trend (Figure 4C). Interestingly, among modulated proteins in pEVs, we found a strong down-regulation of different proteins involved in many processes related to the immune response, such as LG3BP (galectin-3-binding protein), PTX3 (pentraxin 3), CCL5 (C-C motif chemokine 5), ENOA (alpha-enolase), MOES (moesin), and S10A6 (Protein S100-A6). To confirm the results obtained through shotgun MS, we selected 4 proteins according to their immunomodulatory potential and we validated their expression through western blotting. As expected, Galectin-3-binding protein (LG3BP) and pentraxin 3 (PTX3) were up-regulated, while moesin (MOES) and Protein S100-A6 (S10A6) were down-regulated in pEVs compared to cEVs (Figure 4D).

### Protein Annotation and Pathway Enrichment Analysis on Modulated Proteins

**Figure 5:**
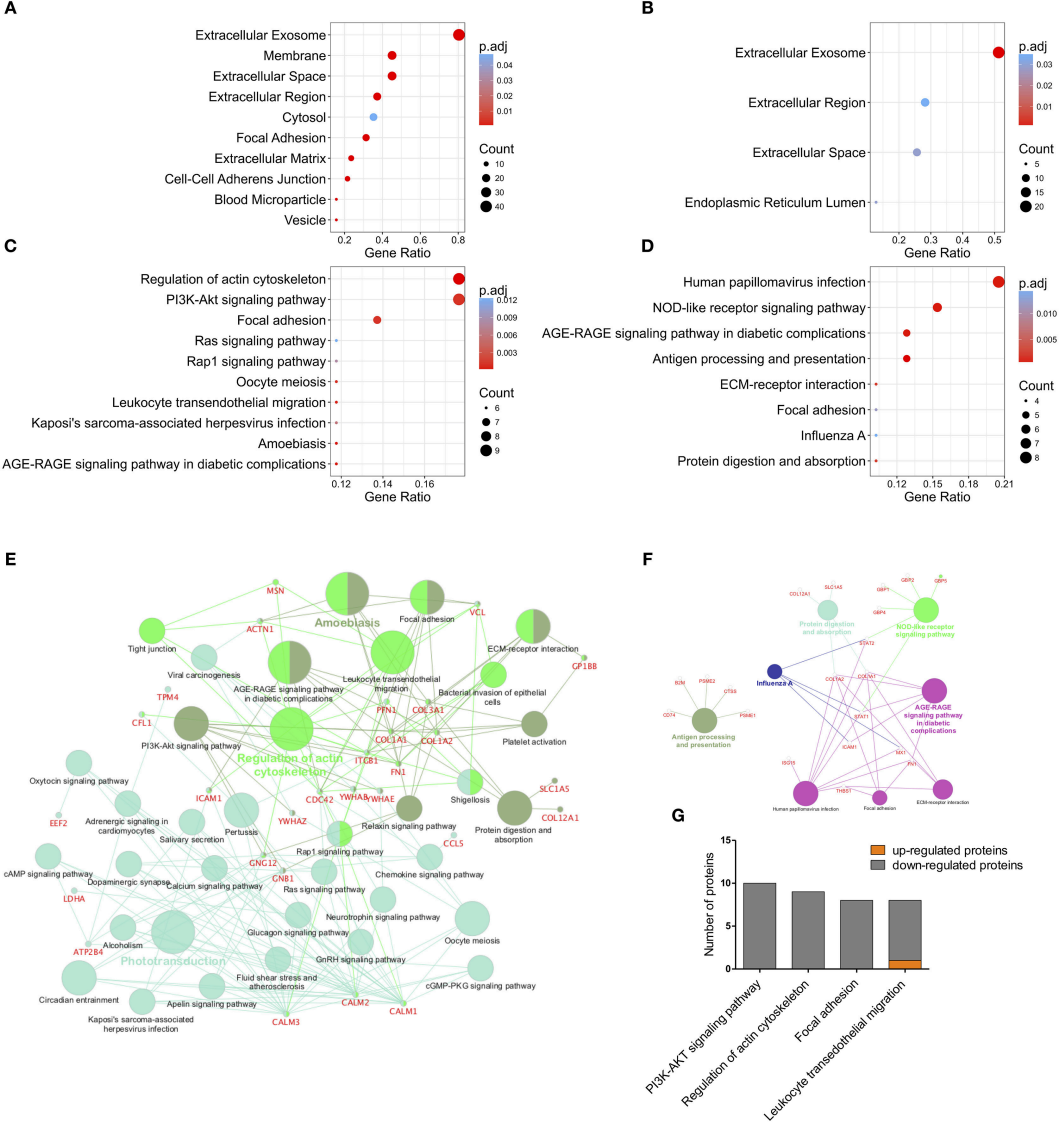
GO protein annotation and Pathway Enrichment Analysis. Cellular component GO category annotation of differentially expressed proteins in pEVs [**(A)**, the top 10 terms are shown] and pMSCs **(B)**. Pathway enrichment analysis on differentially expressed proteins in pEVs [**(C)**, the top 10 terms are shown] and pMSCs **(D)**. Terms with adjusted *p*-value < 0.05 were considered significantly enriched. Cytoscape platform based ClueGO/CluePedia pathway analysis and visualization of differentially expressed proteins in pEVs **(E)** and pMSCs **(F)**. Terms are grouped based on shared genes (kappa score) showed in different colors. The size of nodes indicates the degree of significance. The most significant term defines the name of the group. **(G)** Modulation of differentially expressed proteins in pEVs enriched in “PI3K-AKT signaling pathway,” “regulation of actin cytoskeleton,” “focal adhesion,” and “leukocyte trans-endothelial migration” KEGG pathway (FC < 0.0667 or FC > 1.5, *p* < 0.05).

Differentially expressed proteins of pEVs and pMSCs were annotated on the basis of the GO terms. Considering the pEVs dataset, we found 20 GO terms significantly enriched in the “cellular component” category (Figure 5A), most of which are related to exosomes and EVs, and 11 and 14 terms enriched in “molecular function“ and “biological process” categories, respectively (Figures S2A,C). As for the enriched GO terms of differentially expressed proteins in pMSCs, we found only 4 GO terms in the “cellular component” (Figure 5B), of which the most significant was “extracellular exosome,” only one term in “molecular function” category, and 10 enriched terms in “biological process” category (Figures S2B,D). As expected, many terms refer to biological processes related to the immune system and response to inflammatory stimuli.

To identify potentially perturbed molecular pathways, pEV-derived modulated proteins were mapped to terms in the KEGG database and categorized into 35 pathways significantly enriched (adjusted *p*-value < 0.05) (Figures 5C,E). Notably, the top 10 pathways included the following terms: (i) “PI3K-AKT signaling pathway,” an important pathway involved in cell proliferation, survival, and growth; (ii) “regulation of actin cytoskeleton”; (iii) “focal adhesion”; and (iv) “leukocyte trans-endothelial migration”. These pathways are crucial during leukocyte activation, especially during leukocyte migration through activated venular walls. Modulated proteins resulting enriched in these four KEGG pathways are mainly down-regulated (Figure 5G), thus suggesting a possible inhibition of these pathways in immune effector cells, mediated by pEVs. Pathway enrichment analysis was also performed for deregulated proteins of pMSCs and 8 pathways resulted significantly enriched (Figures 5D,F).

### pEVs Induce a Down-Regulation of PI3K-AKT Signaling Pathway in Activated B Lymphocytes

**Figure 6:**
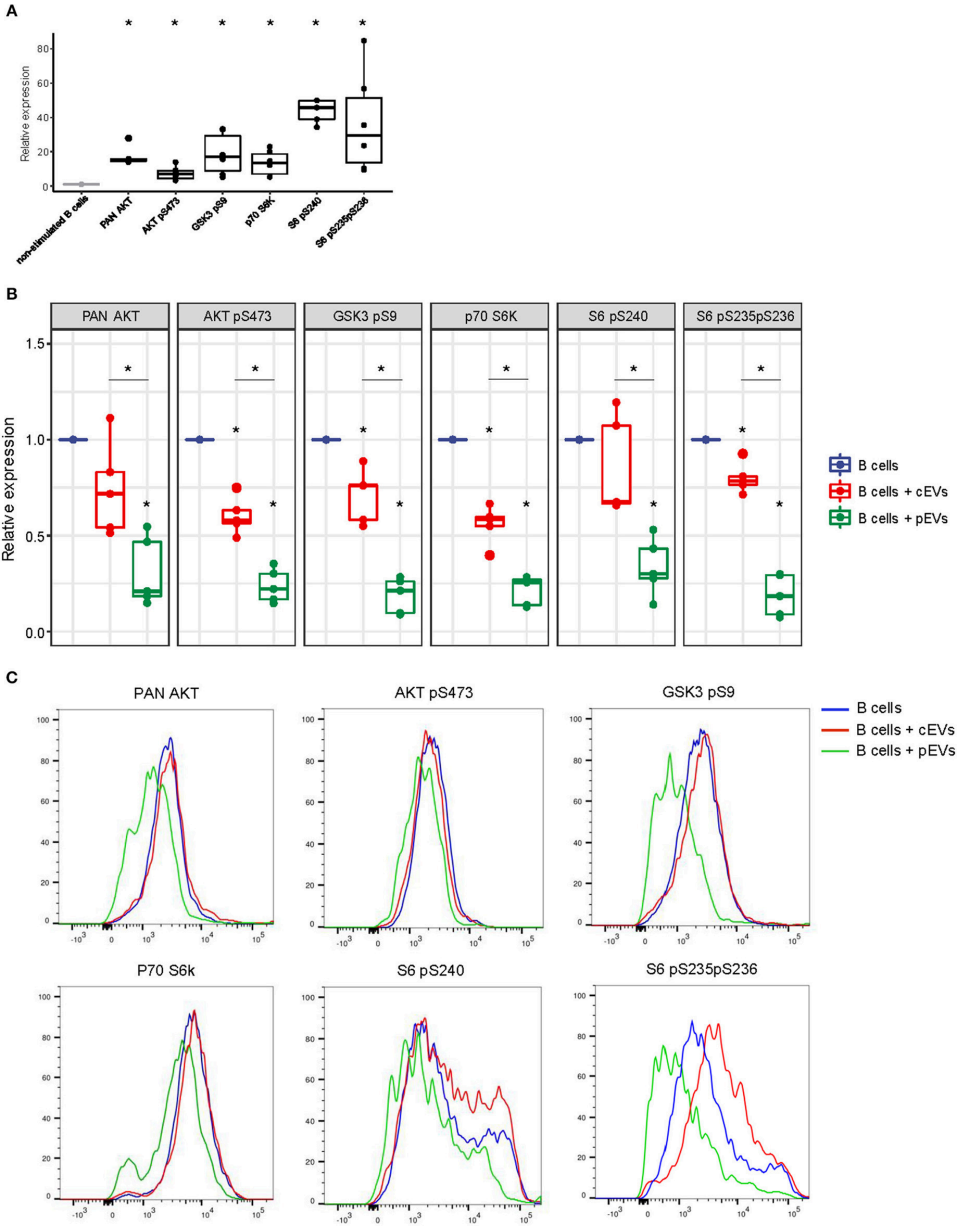
PI3K-AKT signaling pathway expression in activated B lymphocytes co-cultured with MSC-EVs. **(A)** Relative expression of PAN AKT, AKT pS473, GSK3b pS9, p70S6K, S6 pS240, S6 pS235pS236 in activated B lymphocytes analyzed by flow cytometry. **(B)** Relative expression of PAN AKT, AKT pS473, GSK3b pS9, p70S6K, S6 pS240, S6 pS235pS236 in activated B lymphocytes treated or not with resting or primed EVs. Wilcoxon test *p < 0.05. **(C)** Representative histograms showing the levels of expression of PAN AKT, AKT pS473, GSK3b pS9, p70S6K, S6 pS240, S6 pS235pS236 in activated B lymphocytes treated or not with resting or primed EVs.

To evaluate the capability of MSC-derived EVs to modulate the PI3K-AKT signaling pathway, activated B lymphocytes were treated with cEVs or pEVs. The expression and phosphorylation profile of different components of PI3K-AKT signaling pathway (PAN AKT, AKT pS473, GSK3b pS9, p70S6K, S6 pS240, S6 pS235pS236) was evaluated by flow cytometry. We observed a strong up-regulation of the pathway during B cell activation, especially considering the phosphorylation of the ribosomal protein S6, one of the main PI3K-AKT downstream effectors (Figure 6A). As expected, the treatment with pEVs induced a significant down-modulation of the pathway compared to cEVs (Figures 6B,C). We observed a significant reduction of S6 pS235pS236 when activated B lymphocytes were cultured with both cEVs and pEVs. However, the treatment with pEVs led to a significantly higher reduction compared to the treatment with cEVs.

### MSC-Derived EVs Inhibit B Cell Spreading

**Figure 7:**
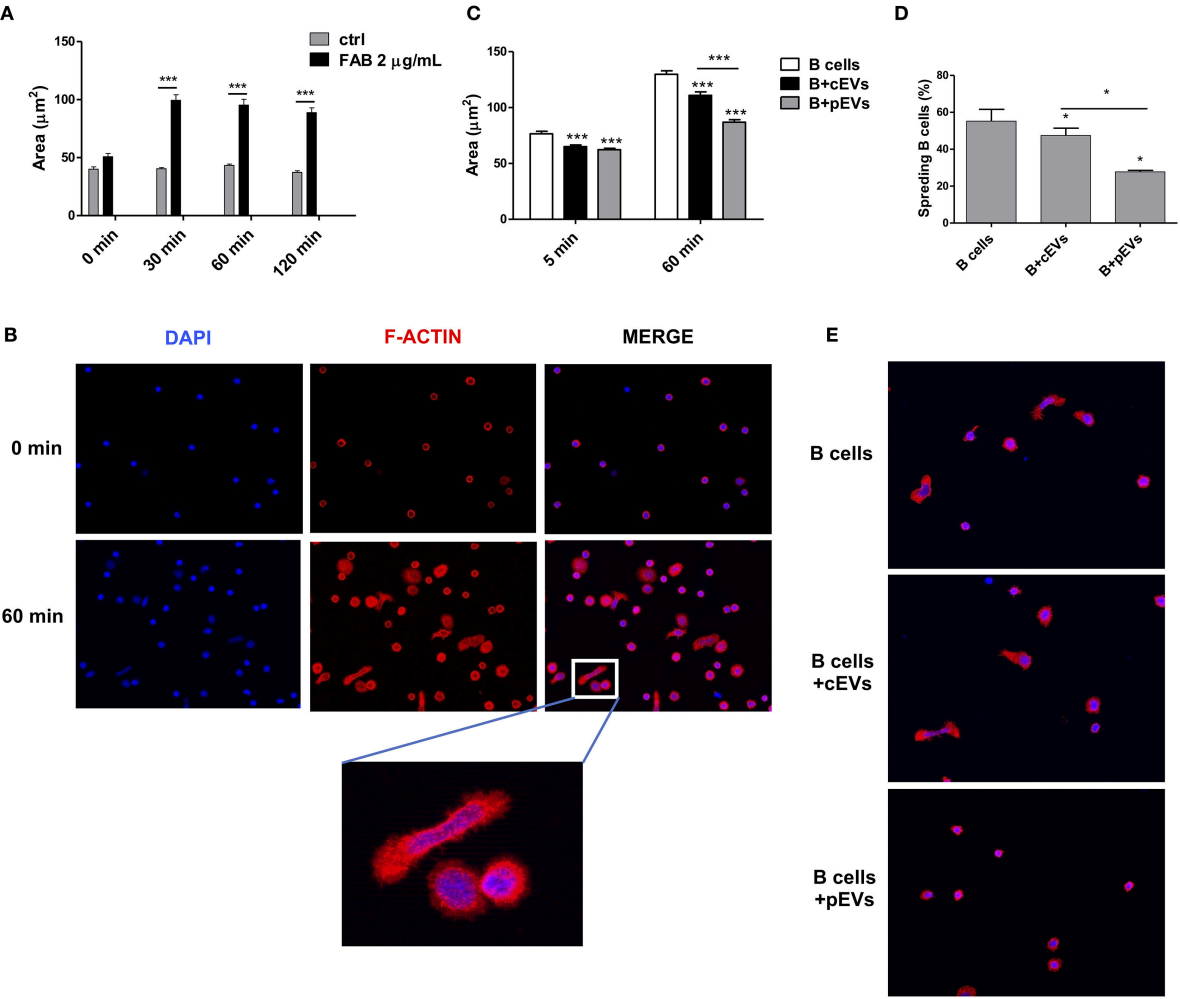
B cell spreading inhibition mediated by MSC-EVs. **(A)** Cell area of B cells plated on coverslips coated or not with F(ab′) 2 anti-human IgM/IgA/IgG (*n* = 42–204). **(B)** Double-stained B cells with DAPI (blue) and rhodamine phalloidin (F-actin, red) before and after the induction of cell spreading (60 min). **(C)** Cell area of B cells pre-treated with cEVs or pEVs and then plated on coated coverslips (*n* = 212–398). **(D)** Percentage of spread B cells treated with cEVs or pEVs after 60 min of incubation on coated coverslips. **(E)** Double-stained B cells with DAPI (blue) and rhodamine phalloidin (F-actin, red) after 60 min of incubation on coated coverslips. The images are representative for three independent experiments with similar results (Original magnification 400x). Error bars represent mean ± SEM of three independent experiments. *t*-test *p < 0.05, ***p < 0.001.

The second most significant KEGG pathway enriched in EV-modulated proteins was “regulation of actin cytoskeleton.” Many works revealed that B cells undergo dramatic morphological reorganization following antigen-mediated activation (50–52). In these mechanisms, the activation of actin cytoskeleton seems to play a crucial role, especially during early events on B cell activation (51). To test the possible role of MSC-derived EVs to promote the reorganization of B cell actin cytoskeleton, we evaluated the ability of B lymphocytes, either pre-treated or not with MSC-derived EVs, to spread on coverslips coated with immobilized F(ab′)2 anti-human IgM/IgA/IgG (53). We first verified the specificity of F(ab′)2 anti-human IgM/IgA/IgG to induce cell spreading (Figures 7A,B). The percentage of cell spreading after 1 h of incubation on coated coverslip was 55%. Spreading cells showed elongated and irregular shape with an average area of 129 ± 59 μm^2^ (Figure 7B). B lymphocytes pre-treated with pEVs showed a significantly reduced cell spreading, with a cell area average of 111 ± 61 μm^2^, as compared to the one treated with cEVs, with a cell area average of 86 ± 41 μm^2^ (Figure 7C). In addition, the treatment with cEVs lowered the percentage of spreading cells to 47%, while spreading cells were strongly reduced to 27% when B lymphocytes were treated with pEVs (Figure 7D) We also observed differences in terms of cell shape in the different experimental conditions. While the morphology of spreading B cells treated with cEVs was similar to the one of the control condition, pEVs induced B cells to retain a spherical shape (Figure 7E). These results confirmed the potential of MSC-derived EVs to modulate the organization of actin cytoskeleton by inhibiting its activation during the processes of B cell spreading. This observation suggests another possible mechanism of action explaining the immunomodulatory properties mediated by MSC-derived EVs, in addition to the modulation of PI3K-AKT signaling pathway.

### miRNA Expression Profile of MSCs and EVs

**Figure 8:**
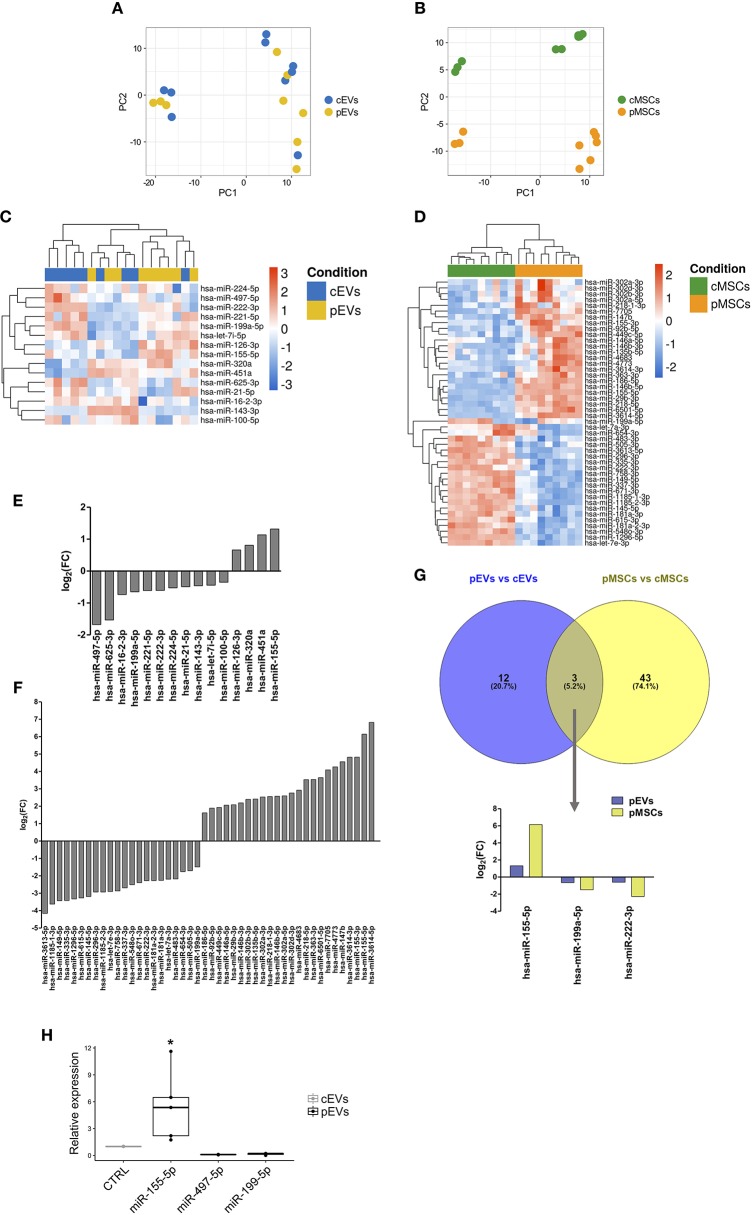
miRNA expression profile of MSCs and EVs following inflammatory priming. PCA plot calculated using regularized-logarithm transformation of miRNA counts on EVs **(A)** and MSCs **(B)** samples. Heatmap summarizing differentially expressed miRNAs in pEVs **(C)** and pMSCs **(D)**. Bar plot representing the log2-fold change of differentially expressed miRNAs in pEVs **(E)** and pMSCs **(F)**. **(G)** Venn diagram representing common differentially expressed miRNAs in pEVs and pMSCs. **(H)** RT-qPCR validation of differentially expressed miRNA in pEVs (*n* ≥ 3). Wilcoxon test *p < 0.05.

To determine miRNA expression pattern in MSCs and EVs following the treatment with inflammatory stimuli, we performed next generation miRNA sequencing. To evaluate the overall variation of the samples, miRNA sequencing data were analyzed by PCA (Figures 8A,B). PCA revealed a clear separation between cMSCs and pMSCc, while cEVs and pEVs were not clearly separated. The same trend was observed using the hierarchical clustering analysis (HCA) among the 9 samples in terms of significantly differentially expressed miRNAs between cMSCs and pMSCs and corresponding EVs (Figures 8C,D). Cellular samples were completely divided into two branches, corresponding to the treated and the control groups, while cEVs and pEVs were not well-separated. These observations highlight that the impact of inflammatory stimuli on miRNA expression is much more evident in MSCs rather than in EVs. Following the treatment with inflammatory stimuli, we found 15 miRNAs differentially expressed in pEVs (Figure 8E), of which 4 up- and 11 down-regulated. Some of them are known to be involved in different immunological responses. In the cellular compartment we found 46 miRNAs differentially expressed between cMSCs and pMSCs (Figure 8F). Three miRNAs resulted modulated with the same trend both in pMSCs and pEVs, i.e., miR-155-5p, miR-199a-5p, and miR-222-3p (Figure 8G).

To assess the functional role of specific miRNAs in MSC-derived EV immunomodulatory properties toward B lymphocytes, we selected miRNAs for RT-qPCR validation by evaluating their experimentally validated targets (Table S5) that were mapped to the terms in the KEGG database. Eventually, miRNA-155-5p, miRNA-199a-5p, and miRNA-497-5p resulted the most promising miRNAs. Table S6 shows the top 20 pathways significantly enriched (adjusted *p*-value < 0.05) for each miRNA. Notably, these miRNAs can modulate the PI3K-AKT signaling pathway, one of the pathway that we found impaired following the treatment with MSC-derived EVs. To confirm the results of miRNA-Sequencing, the expression of miRNA-155-5p, miRNA-199a-5p, and miRNA-497-5p was validated through RT-qPCR (Figure 8H).

### miR-155-5p Reduce B Cell Viability and PI3K-AKT Signaling Pathway

**Figure 9:**
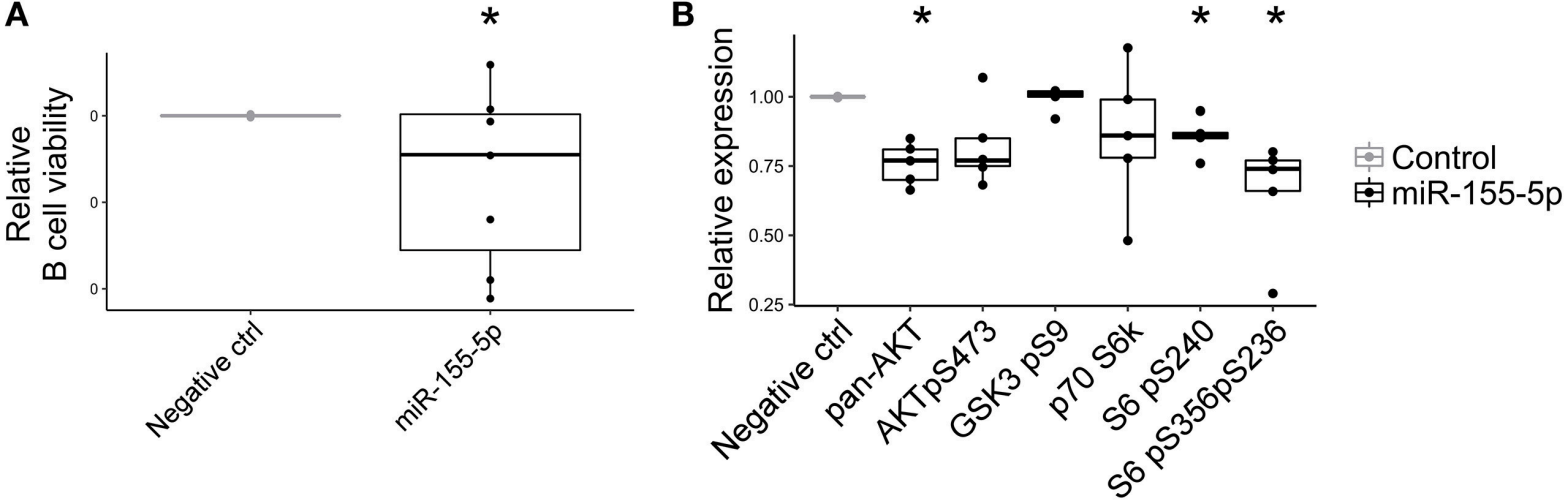
Effect of miR-155-5p on B cell activity. **(A)** Relative cell viability of B cells transfected with double-stranded RNA mimic miR-155-5p (*n* = 7). **(B)** Relative PI3K-AKT signaling pathway expression in B cells transfected with double-stranded RNA mimic miR-155-5p (*n* = 5). Wilcoxon test *p < 0.05.

Due to its strong up-regulation in pEVs and its potential role in immunomodulation processes, we investigated the effect of miR-155-5p on B cell activity. Although the over-expression of miR-155-5p in activated B lymphocytes did not affect cell proliferation (data not shown), we observed a significant reduction in cell viability (Figure 9A). Furthermore, considering that many genes belonging to PI3K-AKT signaling pathway are targeted by miR-155-5p, we assessed its contribution on PI3K-AKT pathway activation. As expected, miR-155-5p contributed to reduce PAN AKT expression and ribosomal protein S6 phosphorylations (Figure 9B).

### miRNA Targets Annotation and Pathway Enrichment Analysis

As the identification of miRNA targets can provide insights into the biological role of differentially expressed miRNA in pEVs and pMSCs, we used miRTarBase, a resource for experimentally validated microRNA-target interactions, to identify the list of experimentally validated miRNA targets (data not shown). To determine their functions, the targets of the top 10 miRNAs modulated in pEVs and pMSCs were annotated on the basis of the GO terms. Figures S3A,B show the top 10 GO terms significantly enriched in the “cellular component,” considering EVs and MSCs compartments, respectively. Interestingly, the enrichment profile resulted almost equal into the two compartments. A similar trend occurred also considering “molecular function” and “biological process” categories (Figures S3C,D,E,F). These observations suggest that probably miRNAs modulated in pEVs only reflected the state of miRNA expression profile of the cell of origin. Many terms that we found in the three GO categories were related to the transcription activity, such as “gene expression,” “RNA binding,” “transcription factor binding,” and “transcription coactivator activity.” Thus, we could expect that the genes targeted by modulated miRNA in pEVs are involved in the regulation of the transcriptional activity of target cells. Concerning the enriched pathways, Figures S3G,H show the top 10 KEGG pathways that could be affected by modulated miRNA in pEVs and pMSCs, respectively. Considering pEVs, we observed a strong enrichment of pathways related to cancer, highlighting a potential role of MSC-derived EVs in mediating cancer-related processes. Moreover, we found the following terms already identified with enrichment analysis on modulated proteins: (i) “focal adhesion,” and (ii) “regulation of actin cytoskeleton.” Thus, the modulation of specific proteins together with the modulation of specific miRNA in pEVs could affect the activity of B lymphocytes, especially during leukocyte migration.

### Combination of miRNA and Proteomic Profiles of MSCs and EVs

**Figure 10:**
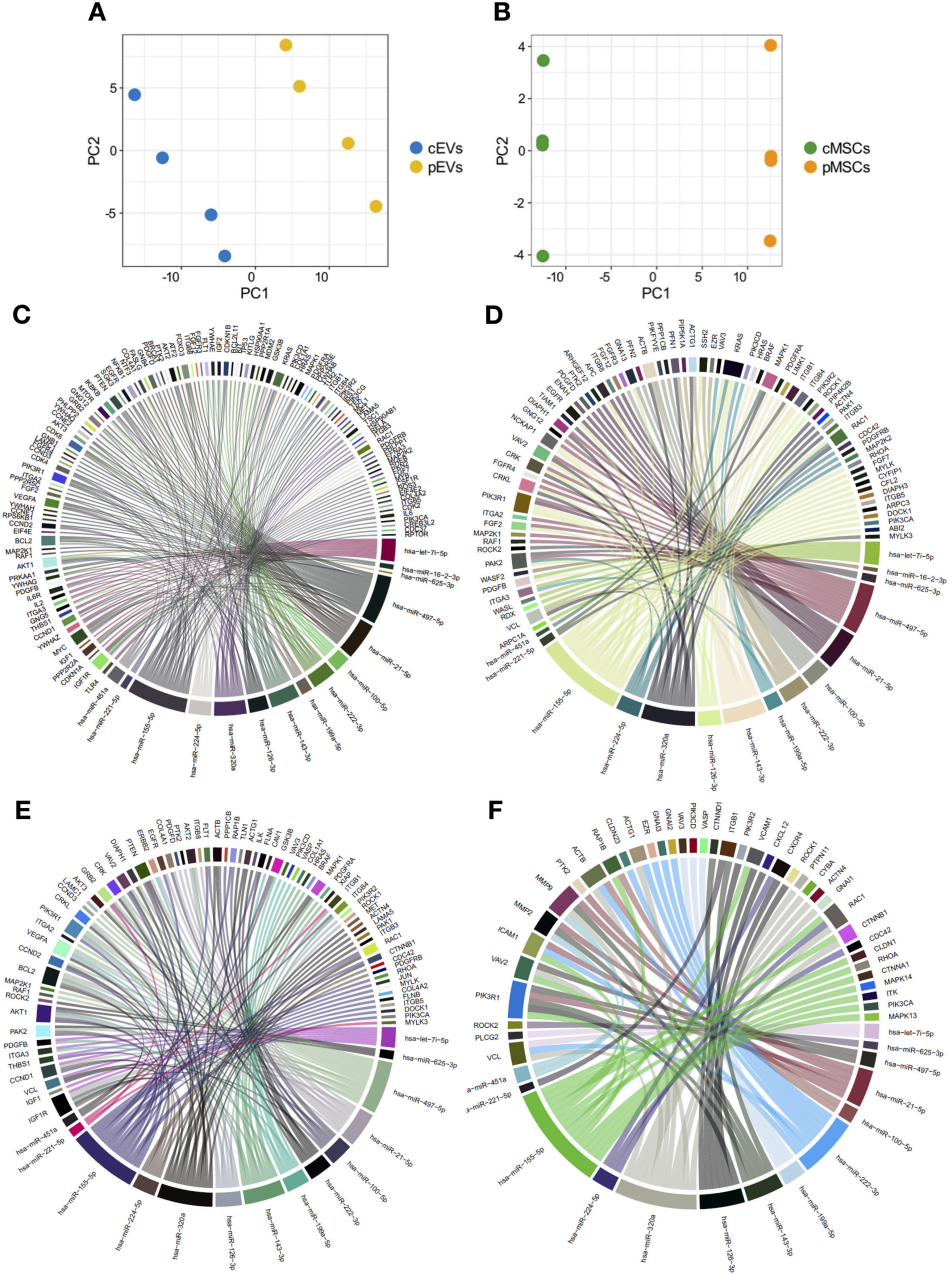
Combination of miRNA and proteomic profiles of MSCs and EVs. Score plot of the first two PCs calculated after the application of PCA to EVs **(A)** and MSCs **(B)**. Chord diagrams representing proteins belonging to “PI3K-AKT signaling pathway” **(C)**, “regulation of actin cytoskeleton” **(D)**, “focal adhesion” **(E)**, and “leukocyte trans-endothelial migration” **(F)** KEGG pathways targeted by miRNAs differentially expressed in pEVs.

To verify possible correlations between miRNA and proteomic profiles, the data from miRNA expression profiles and proteomic determinations were merged by using a multivariate approach. To perform this operation, only the individuals in common with both characterizations were retained; therefore, 4 samples were considered. Only proteins expressed in at least 2 individuals over 4 were considered. Due to the small number of samples involved, only PCA results are presented; however, to show only the correlation structure of the most discriminating variables, PCA was carried out on the first 200 variables selected by ranking-PCA as the most discriminating for both EVs and MSCs. The score plot of the first two PCs calculated for EVs (Figure 10A) and MSCs (Figure 10B) allows the clear separation of the groups of samples.

Looking at the loadings for EVs (Table S7), it is possible to verify that Ranking-PCA first includes mainly miRNA signals (and a lower number of proteins), while the last half of the added signals belongs to the proteomic group. Similar considerations can be drawn from the loadings in the case of MSCs (Table S7); however, in this case Ranking-PCA includes almost exclusively miRNA signals in the first 200 variables and just a few proteins, thus indicating that in the case of MSCs the two characterization profiles are more independent from each other. Positive coefficients correspond to variables (miRNAs or proteins) over-expressed in primed samples, while variables with negative coefficients have the opposite behavior. The loadings show a strong correlation between miRNA profiles and proteomic signals.

### miRNAs Differentially Expressed in pEVs Modulate Pathways Affected by pEV Proteins

To verify other correlations between miRNA and proteomic profiles, we employed an *in-silico* approach to evaluate the possible involvement of miRNAs, differentially expressed in pEVs, in regulating some of the top enriched pathways affected by modulated proteins. We correlated miRNA-Seq and shotgun MS results. To this aim, we evaluated the percentage of genes belonging to “PI3K-AKT signaling pathway,” “regulation of actin cytoskeleton,” “focal adhesion,” and “leukocyte trans-endothelial migration” KEGG pathways targeted by miRNAs differentially expressed in pEVs. All 15 differentially expressed miRNAs targeted more than 50% of genes that constitute “PI3K-AKT signaling pathway” and “regulation of actin cytoskeleton” pathways (61.8 and 52.8%, respectively) (Figures 10C,D). Moreover, 14 miRNAs targeted 58 and 72.4% of genes belonging to “leukocyte trans-endothelial migration” and “focal adhesion” pathways, respectively (Figures 10E,F).

## Discussion

EV-mediated cellular communication has become a hot research topic because of increasing evidence of EV involvement in many physiological and pathological conditions. Depending on their molecular composition, EVs can influence several biological processes by directly activating cell surface receptors and delivering molecular effectors into target cells (11–13). The molecular composition of EVs is strictly influenced by the cell of origin and different environmental conditions; accordingly, EVs secreted by different cell types can modulate the immune system depending on such molecular composition (1, 4, 18, 19).

The predominance of paracrine mechanisms in MSC-mediated immunomodulatory effect has been broadly demonstrated (54–57). These mechanisms include the release of EVs responsible for the reduction of immune effector cell activation, especially as far as B and NK cells are considered (20). MSC priming with inflammatory cytokines dramatically enhances their immunosuppressive properties, thus increasing the capability of EVs they release to reduce the immune responses (20). Furthermore, the immunoregulatory ability mediated by MSCs was comparable to the effect mediated by corresponding EVs, especially when primed-EVs were considered (20). This observation strongly supports the hypothesis that MSC immunosuppressive properties are strictly affected by the EVs they release. Many studies have confirmed the beneficial effect of MSC-derived EVs *in vivo*, paving the way for alternative cell-free therapeutic approaches in the field of inflammatory and autoimmune diseases (58, 59). Nevertheless, the therapeutic application of MSC-derived EVs is still hampered by the lack of standardized and reproducible methods of EV isolation, characterization and quantification and by the difficulty of large-scale production of EVs for therapeutic purposes. In addition, considering their involvement in several physiological and pathological processes, further pre-clinical studies are necessary to exclude possible adverse or simply undesirable effects of MSC-derived EVs. For this reason, a comprehensive analysis of MSC-derived EVs allows to understand the molecular mechanisms underlying their immunomodulatory properties, thus identifying novel therapeutic targets as safer and more useful alternatives to cell or EV-based therapeutic approaches.

On the other hand, the role and prognostic significance of EVs have been documented in a broad range of hematological malignancies, including B-cell leukemia and lymphoma, with a clear involvement in the development and progression of the disease (45–47). In all these pathological conditions, BM stromal cells represent a pivotal player in the survival, proliferation, differentiation, and chemoresistance of neoplastic B cells (60, 61).

Consequently, in this work we focused the analysis on the crosstalk between MSCs and B cells, as we have recently shown that pMSC-derived EVs strongly modulate B cell activity. Here, we showed the effect of inflammatory stimuli on MSC-derived EV secretion and content in terms of miRNA and protein expression profile. We also correlated EVs content with their immunomodulatory properties toward B lymphocytes.

Notably, inflammatory priming induced MSCs to release a higher percentage of exosomes compared to microvesicles, suggesting that the role of smaller vesicles could be functionally more crucial in mediating MSC-related immunosuppressive mechanisms. As shown by flow cytometry, the internalization of cEVs by B lymphocytes resulted higher than pEVs, probably due to the different size of EVs released by MSCs in normal or inflammatory conditions. Inflammatory stimuli affected not only MSC-derived EV size, but also their protein and miRNA content. Protein expression profile of MSCs and MSC-derived EVs was deeply affected by inflammatory priming. Interestingly, modulated proteins in cellular compartment were mostly involved in the release of extracellular exosomes, thus highlighting the significance of EV release by MSCs during inflammatory processes and, consequently, their role in MSC-mediated immunoregulation. Furthermore, modulated proteins in pEVs are involved in different processes related to immunological events, especially during leukocyte activation and migration. Such processes include “PI3K-AKT signaling pathway” and “regulation of actin cytoskeleton.” PI3K-AKT signaling pathway is an intracellular pathway affecting the function of several biological processes, such as cell survival, cell cycle progression, and cellular growth (62), while the regulation of actin cytoskeleton is pivotal during leukocyte activation, especially during the early stage of B cell receptor activation (50). The proteins belonging to these pathways resulted down-modulated, thus suggesting their possible involvement during the process of immunosuppression toward effector cells. As expected, pEVs induced a strong down-modulation of PI3K-AKT components and a substantial impairment of actin reorganization in B lymphocytes during the early stages of activation. These results suggest some of the potential mechanisms of action underlying the immunomodulatory properties mediated by MSC-derived EVs. Pathway enrichment analysis for modulated proteins in pMSCs revealed only four terms, including “antigen processing and presentation” pathway as significantly enriched, highlighting the well-known capacity of MSCs to present antigen via major histocompatibility complex (MHC) molecules, which are constitutively expressed and up-regulated on MSCs following inflammatory priming (20, 63). Similarly, only the term “identical protein binding” resulted significantly enriched in the molecular function GO category. Taken together, our results showed that inflammatory stimuli mainly affect the protein content of EVs compared to the originating cells, confirming their crucial role and the predominance of paracrine mechanisms in mediating immunomodulatory functions. Focusing on modulated protein in pEVs, we found several proteins involved in many processes related to immune response, such as LG3BP, PTX3, MOES, and S10A6. Galectins are a family of beta-galactoside-binding proteins implicated in modulating cell-cell and cell-matrix interactions. LG3BP promotes integrin-mediated cell adhesion and influences different processes of immune response, such as NK cell activation and lymphokine-activated killer (LAK) cell cytotoxicity (64). PTX3 is involved in the regulation of innate resistance to pathogens and inflammatory reactions. The expression of this protein is induced by inflammatory cytokines in several mesenchymal and epithelial cell types (65). MOES and S10A6 are also involved in immune response mechanisms, especially regarding the organization of actin cytoskeleton and cell motility (66–68). Furthermore, the Ezrin, Radixin, and Moesin (ERM) protein family is not only involved in the cytoskeletal reorganization; such proteins are also involved in the transmission of signals in response to different extracellular stimuli through their ability to crosslink transmembrane receptors with downstream signaling components in B cells (68). Considering their function, each of such modulated protein could contribute to immunoregulatory activity mediated by MSC-derived EVs. In addition, pEVs showed a strong up-regulation of ITI components. ITI represents a family of structurally related plasma serine protease inhibitors involved in extracellular matrix stabilization (69). Frequent loss of expression of ITIH genes is recurring in multiple human solid tumors, thus promoting tumor metastasis (70). This observation further strengthens the role of MSC-derived EVs in cancer, with a potential therapeutic action in preventing tumor metastasis.

Inflammatory microenvironment affected not only MSC-derived EVs proteins, but also miRNA expression. In consideration of miRNA stability and involvement in modulating gene expression, their role in cell-to-cell communication mediated by EVs has been increasingly studied in the last years. As for miRNA overall variation following inflammatory priming, enriched GO terms resulted similar in pMSCs and pEVs, showing less remarkable differences between cellular and EV compartment in miRNA composition as compared to proteins composition. Modulated miRNAs influence many processes related to transcription activity; accordingly, pathway enrichment analysis showed a strong enrichment of pathways related to cancer in both dataset. On the other hand, there is a clear evidence that MSCs and MSC-derived EVs can enhance or suppress tumor progression depending on tumor model and stage considered (71–73). Nevertheless, differentially expressed miRNAs in pEVs are also involved in immunomodulatory processes. Among these, we identified “focal adhesion” and “regulation of actin cytoskeleton” KEGG pathways, already found amongst modulated proteins. Thus, the modulation of specific proteins together with the modulation of specific miRNAs in pEVs could affect the activity of immune effector cells, especially during leukocyte migration.

Protein and miRNA molecules contained in MSC-derived EVs following inflammatory priming are strictly connected. They could be transferred into target cells and contribute synergistically to the regulation of immune effector cell activity. Notably, we found that all differentially expressed miRNAs in pEVs influence the most significant enriched pathways resulting from the protein differential expression analysis. Furthermore, many of them have a crucial role in immunological processes in different cellular contexts (74). In particular, miRNA-155-5p is one of the most well-characterized miRNA regulating immune response (75–77). Here, we showed that miRNA-155-5p has a direct capability to reduce cell viability in activated B lymphocytes. This effect could be in part mediated by its ability to act as negative regulator of PI3K/AKT signaling pathway, one of the pathways we proved to be impaired following the treatment with MSC-derived EVs.

In summary, our data show that MSC-derived EVs contain several molecules potentially responsible for their immunomodulatory properties and potentially involved in MSC/B-cell crosstalk; in particular, specific molecules modulated in pEVs are capable of influencing typical immune effector cell activity, thus representing novel potential therapeutic targets that could be further investigated in the field of inflammatory and autoimmune diseases as well as in neoplastic microenvironment of B-cell malignancies.

## Materials and Methods

### Cell Culture

MSCs were isolated from BM aspirates of healthy donors (informed consent, approved by Ethical Committee of Azienda Ospedaliera Universitaria Integrata Verona; N. 1828, May 12, 2010 “Institution of cell and tissue collection for biomedical research in Onco-Hematology”) and characterized as already described (78, 79). MSCs were cultured in DMEM supplemented with 10% heat-inactivated fetal bovine serum (FBS), 1% penicillin-streptomycin, and 2% L-Glutamine (all from Sigma-Aldrich). All experiments were performed between passages 2 and 7. MSCs at 80% confluence were treated or not for 48 h with 10 ng/mL IFN-γ and 15 ng/mL TNF-α (R&D Systems) to induce inflammatory priming. PBMCs were isolated from human blood using Lymphoprep (Stemcells Technologies). B lymphocytes were isolated from PBMCs using immunomagnetic negative selection (Miltenyi Biotec) with at least 95% cell purity, as evaluated by flow cytometry (78, 79). B lymphocytes were cultured in RPMI medium supplemented with 10% FBS, 1% penicillin-streptomycin and 2% L-Glutamine (all from Sigma-Aldrich). B lymphocytes were activated with 5 μg/mL anti-human IgM+IgA+IgG (F(ab′)2, Jackson Immunoresearch), 50 IU/mL rhIL-2 (Novartis), 50 ng/mL polyhistidine-tagged CD40 ligand, 5 μg/mL anti-polyhistidine antibody (R&D Systems), and 0.5 μg/mL CpG ODNs (Invitrogen). The proliferation was assessed by flow cytometry on viable TOPRO-3^neg^ CD45^pos^ cells by using FlowJo software (TreeStar) as the percentage of cells undergoing at least one cell division. B cells and MSC were co-cultured in allogeneic setting.

### Purification, Characterization and Quantification of MSC-Derived EVs

EVs were isolated from conditioned medium by ultracentrifugation as already described. EVs quantification was performed through BCA Protein Assay (Thermo Fisher Scientific). Particle size was evaluated by dynamic light scattering (DLS) measurements using a Zetasizer Nano ZS (Malvern Instruments, 4 mV He-Ne laser, λ_0_ = 633 nm, θ = 173°). To assess the surface marker profile, 10 μg of EVs were analyzed using MACSPlex Exosome Kit (Miltenyi Biotec), a multiplex bead-based platform that allows the detection of 37 surface epitopes. Samples were analyzed with a FACS Canto II (BD Bioscences). Mean fluorescence values were background corrected according to the protocol. *In vitro* experiments with MSC-EVs were carried out using pooled EVs derived from at least 5 MSC donors, with the aim to reduce interindividual variability.

### EV Internalization and Fluorescence Microscopy

To assess EV internalization by B lymphocytes, MSC membranes and RNA were stained with 200 μM Vybrant Dil Cell-labeling Solution and 500 nM Syto RNA Select (both from Life Technologies), respectively. Then, labeled or unlabeled MSCs were co-cultured in presence of B lymphocytes (1:1) and EV internalization was assessed after 24, 48, and 72 h. At the end of the co-culture, cells were stained with anti-CD45-PerCP-Vio770 (Miltenyi Biotec) and TOPRO-3 Iodide (Life Technologies), allowing us to exclude MSCs from our analyses performed on CD45 positive viable cells by flow cytometry. Furthermore, EVs were directly stained with 10 μM Vybrant Dil Cell-labeling Solution and 10 μM Syto RNA Select and washed with Exosome Spin Column (MW 3000) (Thermo Fisher Scientific). Then, activated B lymphocytes were cultured for 24 h with of labeled or unlabeled EVs (30 μg/2 × 10^4^ B cells). EVs internalization were evaluated by flow cytometry using the same gating strategy used for MSC/B cell co-cultures. For fluorescence microscopy experiments, B cells were loaded into the CytoSpin centrifuge's sample chamber and centrifuged 300xg for 5 min. Finally, cells were stained with 1 μg/mL Hoechst (Thermo Fisher) in PBS and then analyzed by fluorescence microscopy using a Zeiss Axio Observer Z1 microscope (Carl Zeiss).

### Sample Preparation for Shotgun Proteomics

EVs were collected and lysed in PBS supplemented with protease inhibitors (Roche) and 1% Sodium Dodecyl Sulfate (SDS) (Bio-Rad). Protein extraction was performed by 5–6 cycles of sonication. Then, ice-cold acetone was added to samples and protein precipitation was conducted overnight at −20°C. Samples were then centrifuged at 14,000xg for 10 min at 4°C, and pellet was resuspended in 100 mM NH_4_HCO_3_. Protein concentration was measured with BCA Protein Assay. Samples were subjected to denaturation with trifluoroethanol, reduced with DTT 200 mM, alkylation with Iodoacetamide 200 mM, and digested with Trypsin/Lys-C (Promega). The peptide digests were desalted on the Discovery® DSC-18 solid phase extraction (SPE) 96-well Plate. The SPE plate was preconditioned with 1 ml of acetonitrile and 2 ml of water. After the sample loading, the SPE was washed with 1 ml of water. The adsorbed proteins were eluted with 800 μl of acetonitrile:water (80:20). After the desalting, the sample was vacuum-evaporated and reconstituted with 20 μl of 0.05% formic acid in water before LC-MS/MS analysis.

### Shotgun Mass Spectrometry

The digested proteins were analyzed with a micro-LC Eksigent Technologies (Eksigent) system interfaced with a 5600+ TripleTOF system (AB Sciex) equipped with DuoSpray Ion Source and Calibrant Delivery System. More detailed information on instrument setting and label-free quantification is available in Supplementary Materials and Methods. Two DDA and three DIA acquisitions were performed (80).

The DDA files were searched using Protein Pilot software v. 4.2 and Mascot v. 2.4. Trypsin/Lys-C as digestion enzyme was specified for both the software. For Mascot we used 2 missed cleavages, the instrument was set to ESI-QUAD-TOF and the following modifications were specified for the search: carbamidomethyl cysteines as fixed modification and oxidized methionine as variable modification. A search tolerance of 50 ppm was specified for the peptide mass tolerance, and 0.1 Da for the MS/MS tolerance. The charges of the peptides to search for were set to 2 +, 3 +, and 4 +, and the search was set on monoisotopic mass (81, 82).

The UniProt Swiss-Prot reviewed database containing human proteins (version 2015.07.07, containing 42131 sequence entries) was used and a target-decoy database search was performed. False Discovery Rate was fixed at 1%.

The protein quantification was performed by integrating the extracted ion chromatogram of all the unique ions for a given peptide and was carried out with PeakView 2.0 and MarkerView 1.2. Six peptides per protein and six transitions per peptide were extracted from the SWATH files. Shared peptides were excluded as well as peptides with modifications. Peptides with FDR lower than 1.0% were exported in MarkerView for the *t*-test (83).

Proteomic *t*-test statistical analysis was performed with MarkerView. Proteins were considered modulated in presence of a fold change ≥ 1.50 or ≤ 0.5 and a *p*-value < 0.05.

### Western Blot Analysis

Immunoblot analysis on EV candidate proteins was performed as previously described (84). Briefly, protein samples from different biological replicates were diluted 1:1 with Laemmli's sample buffer (62.5 mM Tris-HCl, pH 6.8, 25% glycerol, 2% SDS, 0.01% Bromophenol Blue), heated for 5 min at 90°C, and separated by SDS/polyacrylamide gel electrophoresis (PAGE) on 4–20% T acrylamide gels in Tris/glycine/SDS buffer. After separation on SDS-PAGE, proteins were electroblotted on PVDF membrane and subjected to immunodetection. Amido Black staining (Sigma-Aldrich) was used to confirm equal protein loading in different lanes. Membranes were incubated with the different primary antibodies diluted in 1% non-fat dried milk, 0.05% Tween-20 in Tris-buffered saline for 3 h at room temperature. Blots were then incubated 1 h at room temperature with the appropriate horseradish peroxidase (HRP)-conjugated secondary antibody (see Table S8). Immunocomplexes were visualized by chemiluminescence using the Chemidoc MP imaging system (Bio-Rad) and the intensity of the chemiluminescence signal was measured by processing the image with Image Lab software (Bio-Rad).

### Univariate and Multivariate Statistical Analysis

Proteomic data were analyzed by univariate and multivariate statistical analyses. The univariate approach consisted in selecting proteins modulated at least in 4 samples among 7 processed samples using a cut off with adjusted *p*-values of <0.05 and fold change of >1.5 or <0.0667. Principal Component Analysis (PCA) was applied to provide a general overview of the correlations existing between the variables and the existence of sample groups (see Supplementary Materials and Methods). PCA is exploited in Ranking-PCA to select the most discriminating variables (i.e., candidate biomarkers) between two groups of samples and sort them according to their decreasing discrimination ability (85–88).

PLS is a multivariate regression method establishing a relationship between one or more dependent variables (Y) and a group of descriptors (X). X and Y variables are modeled simultaneously, to find the latent variables (LVs) in X that will predict the LVs in Y. PLS can be applied for classification purposes by establishing an appropriate Y related to the association of each sample to a class. Since two classes are present in this case (control and primed samples), a binary Y variable is added to each dataset, coded so that−1 is attributed to control samples and +1 to primed samples. The regression is then carried out between X-block variables (protein counts) and the Y just established. This application for classification purposes is called PLS-DA (89). Here, Ranking-PCA was used as selection method to provide the list of all candidate biomarkers in decreasing order of discriminant ability of the final model. The list identified in this way was then used for classification, based on PLS-DA (89), to provide a final model able to discriminate the two classes of samples present. The analysis was done considering 1 PC in Ranking-PCA and 1 LV in the final PLS-DA models (for MSCs, only the first 200 proteins selected by Ranking-PCA were included in the PLS-DA model). Both the selection of the significant proteins by Ranking-PCA and the calculation of the performance ability of the PLS-DA models were obtained by leave-one-out (LOO) cross-validation: all the replications of the same sample (both control and primed replications) were eliminated in turn from the calculation and used to provide an evaluation of the final predictive ability. Since 7 individuals were available, only LOO was applied, and one individual was eliminated at a time. In Ranking-PCA just one PC was used for calculation, due to the small number of degrees of freedom available. The classification performances were evaluated by calculating model accuracy, i.e., the ratio of correctly assigned samples, and the Non-Error-Rate% (NER%), i.e., the average of the accuracies calculated for each class independently.

### RNA Extraction and Quality Controls

Total RNA was extracted using miRNeasy Mini Kit (Qiagen) and miRNA enriched fraction was obtained using mirPremier microRNA Isolation Kit (Sigma Aldrich) according to manufacturer's instructions. RNA was quantified with the Qubit RNA Assay kit (Life Technologies). RNA purity and integrity were assessed at the Nanodrop 1000 spectrophotometer (Thermo Fisher Scientific) and by capillary electrophoresis on an Agilent Bioanalyzer (Agilent Technologies), respectively.

### Small RNA Sequencing

Small RNA libraries were prepared using the NebNext Small RNA Library Preparation Kit (New England Biolabs) for miRNA and short non-coding RNAs sequencing according to manufacturer's instructions, starting from 200 ng RNA and using 15 PCR cycles. Each library was analyzed with the High Sensitivity DNA chip (Agilent) using Agilent 2100 Bioanalyzer. Pools of 12 libraries were concentrated with AMPure XP magnetic beads (Beckman Coulter) and eluted in 40 μl of nuclease free water. The eluate from each pool was loaded onto a 6% precast gel (Life Technologies), a ≈146 bp band was cutted, purified with a 5 μl filter with Qiaquick gel extraction kit (Qiagen) and quantified using the Qubit DNA HS (Life Technologies). The miRNAseq libraries were sequenced on a NextSeq500 sequencer (Illumina) using 75 bp-reads and producing on average 2,047,034,738 reads per sample.

### Identification of Differentially Expressed miRNAs

Illumina sequencing was used to generate small RNA reads from 9 biological replicates of primed and control EVs/MSCs. Raw fastq files were analyzed to evaluate their quality using FastQC software (http://www.bioinformatics.babraham.ac.uk/projects/fastqc). Adapter trimming was performed using TrimGalore (https://www.bioinformatics.babraham.ac.uk/projects/trim_galore) specifying *length* and *max-length* parameters. After this step, only reads with length between 16 and 26 nucleotides remained for further analysis. Clipped reads were aligned using Bowtie2 to the GRCh38 human genome built from ENSEMBL, using the preset of parameters –*very-sensitive-local* to obtain high-score matches (90). The summarization of the reads was performed using featureCounts (91), using the GFF annotation file provided by miRBase. Differential expression analysis was conducted through the R/Bioconductor package DESeq2 comparing primed vs. control EV/MSC samples, using a 5% FDR cut-off (92).

### Real-Time qPCR

For miRNA analysis, 10 ng of RNA was retro-transcribed with TaqMan MicroRNA Reverse Transcription Kit (Applied Biosystems). Relative miRNA expression was determined using TaqMan Universal Master Mix II, no UNG and Taqman microRNA assay (Applied Biosystems) and normalized to U6. All reactions were performed in triplicates and run on LightCycler 480 Instrument II (Roche) following manufacturer's instructions. Data obtained from the qPCR were analyzed using the ΔΔCt method.

### GO Annotation and Pathway Enrichment Analysis of Differentially Expressed Proteins

GO annotation of significantly altered proteins expressed in pEVs was performed with DAVID v6.8 Functional Annotation Bioinformatics Tools (https//david.ncifcrf.gov/). The enrichment analysis for GO cellular component, biological process and molecular function was done by comparing the GO terms of identified proteins against the human proteome. This analysis detected the significant (p adj < 0.05) over-representation of GO terms in the submitted dataset. In addition, ClueGO v2.3.3, was used for analyzing significantly enriched KEGG pathways (p adj < 0.05) with the human genome as background (93). The nodes in functionally grouped networks were linked based on their kappa score level (0.4) in ClueGO; the GO Tree Levels ranged from levels 3–8.

### GO Annotation and Pathway Enrichment Analysis of Modulated miRNAs and miRNA Targets

Functional enrichment of top 10 (based on adjusted *p*-value) differentially expressed miRNAs in pEVs and pMSCs was performed using DIANA-miRPath v3.0 (94). The enrichment analysis was conducted on KEGG and GO using only experimentally validated miRNA-target interactions provided by DIANA-TarBase v7.0, with *genes union* as merging algorithm. Furthermore, the parameter *conservative stats* were used to have a more stringent statistical test. Only terms with an adjusted *p*-value < 0.01 were selected.

### *In vitro* Cell Spreading

Glass coverslips were coated at room temperature for 2 h with 5 μg/mL of F(ab′)2. 15x104 B lymphocytes, pre-treated for 24 h with resting, and primed EVs, were left to spread at 37°C on coverslips for different time points. Then, cells were fixed with 4% paraformaldehyde in PBS (ChemCruz), permeabilized with 0.5% Triton X-100 (AppliChem) and stained with DAPI and rhodamine phalloidin (both from Life Technologies). Cell spreading was quantified by measuring the mean cell area (μm2) by using AxioVision 4.8.2 software (Carl Zeiss).

### miRNA Mimic Transient Transfection

Activated B cells were seeded at 2 × 105/200 μL per well. After 24 h, cells were transfected with 10 nM of miR-155-5p mimic (5′UUAAUGCUAAUCGUGAU AGGGGU) or negative control miRNA (5′GAUGGCAUU CGAUCAGUUCUA) both from Exiqon using INTERFERin (Polyplus-transfection S.A.). After 6 h, complete RPMI medium supplemented with stimuli was added to each well. Finally, cells were harvested for the functional analyses after 24, 96, or 120 h.

### PI3K/AKT Signaling Pathway Expression Profile

B lymphocytes were cultured for 4 days in presence or absence of EVs (30 μg EVs/2x104 cells) or transfected with miRNA Mimics. At the end of culture, cells were harvested and stained with CD45-PerCpVio700 and Viobility 405/520 Fixable Dye (both from Miltenyi Biotec). Then, cells were fixed and permeabilized using Cell Signaling Buffer Set A (Miltenyi Biotec) and stained with Anti-AKT pS473-Vio515, Anti-AKT Pan (PKB)-APC, Anti-GSK3B pS9-PE, Anti-p70 S6 Kinase-FITC, Anti-S6 pS235/pS236-PE, and Anti-S6 pS240-APC (all from Miltenyi Biotec) according to manufacturer's instructions. Protein expression was assessed by flow cytometry. Data analysis was performed using FlowJo software (TreeStar). Data were expressed as mean ± SEM. Statistical analysis was performed by Pism software (GraphPad) using the Wilcoxon test. *P* < 0.05 was considered statistically significant.

### Cell Proliferation and Viability Assay

To evaluate the effect of selected miRNAs on B cell proliferation, cells were activated and stained with 5 μM carboxyfluorescein succinimidyl ester (CFSE, Life Technologies). After 24 h, cells were transfected with miRNA mimics and negative control. After 4 days, cells were harvested and stained with anti-human CD45-PerCP-Vio700 (Milteniy Biotec) and TOPRO-3 Iodide (Life Technologies). The effect of selected miRNA on cell viability was assessed by flow cytometry using AnnexinV/7AAD staining. Following 24 h stimulation, cells were transfected with miRNA mimics and negative control. After 24 h, cells were harvested and stained with anti-human CD45-APC (Milteniy Biotec), Annexin V-FITC (Milteniy Biotec) and 7AAD (BD Biosciences). Cell viability was evaluated on CD45^pos^Annexin V^neg^ 7AAD^neg^ cells using FlowJo software (TreeStar). Data were expressed as mean ± SEM. Statistical analysis was performed by Prism software (GraphPad) using the Wilcoxon test. P < 0.05 was considered statistically significant.

## Data Availability

The datasets produced in this study are available at the following link:

https://www.dropbox.com/sh/prf3r38hmdrr08g/AAAczrn_rSvLkYWbVQK9cySUa?dl=0.

## Author Contributions

AA designed and performed the laboratory work and wrote the manuscript. JB performed the laboratory work and contributed to paper writing. RB, RC, MM, GB, PT, GD, AG, AM, MA, and MO performed the laboratory work. DC contributed to paper writing. SC, PD, RG, ER, EM, and RAC performed statistical analysis and contributed to paper writing. MK coordinated the research plan, wrote and approved the final version of the paper.

### Conflict of Interest Statement

The authors declare that the research was conducted in the absence of any commercial or financial relationships that could be construed as a potential conflict of interest.
