# Supplementary material for: Extracellular Vesicles Mediate Mesenchymal Stromal Cell-Dependent Regulation of B Cell PI3K-AKT Signaling Pathway and Actin Cytoskeleton
Source: Front Immunol. 2019 Mar 12;10:446. doi: 10.3389/fimmu.2019.00446 (PMC6423067; doi:10.3389/fimmu.2019.00446)
Supplement: Supplementary file 2 [file Table_2.DOCX]

| *Mesenchymal Stromal Cells* | | | | | | | | |  |
| --- | --- | --- | --- | --- | --- | --- | --- | --- | --- |
|  | Identified proteins | | | Modulated proteins (pMSCs vs cMSCs) | | | | |  |
|  | cMSCs | pMSCs | FC>1.5 | | | FC<0.667 | | |  |
| D24 | 1154 | 951 | 69 | | | 46 | | |  |
| D21 | 663 | 857 | 60 | | | 33 | | |  |
| D8 | 757 | 807 | 47 | | | 49 | | |  |
| D29 | 821 | 829 | 64 | | | 111 | | |  |
| BM004 | 643 | 581 | 54 | | | 120 | | |  |
| D33 | 856 | 1136 | 179 | | | 77 | | |  |
| D36 | 1168 | 887 | 70 | | | 154 | | |  |
|  |  |  | | |  | |  | | |
| *Extracellular Vesicles* | | | | | | | | |  |
|  | Identified proteins | | | Modulated proteins (pEVs vs cEVs) | | | | |  |
|  | cEVs | pEVs | FC>1.5 | | | FC<0.667 | |  |  |
| D24 | 423 | 302 | 110 | | | 113 | |  |  |
| D21 | 203 | 165 | 18 | | | 54 | |  |  |
| D8 | 100 | 138 | 58 | | | 9 | |  |  |
| D29 | 414 | 363 | 21 | | | 53 | |  |  |
| BM004 | 230 | 216 | 49 | | | 78 | |  |  |
| D33 | 432 | 281 | 46 | | | 172 | |  |  |
| D36 | 392 | 282 | 23 | | | 115 | |  |  |

**Supplementary information, Table S2.** Identified and modulated proteins in resting and primed MSCs and corresponding EVs using shotgun MS.
