# Supplementary material for: Extracellular Vesicles Mediate Mesenchymal Stromal Cell-Dependent Regulation of B Cell PI3K-AKT Signaling Pathway and Actin Cytoskeleton
Source: Front Immunol. 2019 Mar 12;10:446. doi: 10.3389/fimmu.2019.00446 (PMC6423067; doi:10.3389/fimmu.2019.00446)
Supplement: Supplementary file 5 [file Table_5.DOCX]

**Supplemetary information, Table S5.** Experimentally validated target genes of miRNA miR-155-5p, miR-497-5p and miR-199a-5p.

| miRNA ID | Experimentally validated miRNA targets |
| --- | --- |
| hsa-miR-155-5p | MEIS1,TAB2, MECP2, SOCS1, MSH6, MSH2, MLH1, INPP5D, DET1, SMAD5, HIVEP2, ZNF652, ZIC3, BACH1, JARID2, APC, WDFY1, VAMP3, UBE2J1, TXNRD1, TXNDC12, TRIP13, TRIM32, TRAM1, TNFRSF10A, TBCA, TACSTD2, SYPL1, SYNE2, SNAP29, SMAD1, SLC30A1, SH3BP4, SDCBP, SCAMP1, RHEB, RCOR1, RCN2, RAI14, RAB6A, RAB5C, RAB34, RAB27B, RAB23, PTPRJ, PRKCI, PRAF2, PPP5C, PPL, POLE4, POLE3, PODXL, PLXND1, PKN2, PICALM, PHC2, PDLIM5, PDE3A, NT5E, NARS, MYO1E, MYO10, MSI2, MPZL1, MOSPD2, MARC1, METTL7A, LY6K, LPL, UFL1, HSDL1, HSD17B12, GNA13, FMNL2, FADS1, DSG2, DPP7, DNAJC19, DNAJB1, DHX40, CYP51A1, CUL4B, CTNNB1, CLDN1, CHAF1A, CEBPB, CDK5RAP3, CBFB, BRPF3, BET1, ATP6V1C1, ATG3, ARL5B, ARL10, ARID2, ARFIP2, ARFIP1, ANKFY1, AMIGO2, TM6SF1, MATR3, LDOC1, JADE1, RHOA, AGTR1, TP53INP1, FGF7, IKBKE, NFATC2IP, CUX1, BCAT1, SPI1, CTLA4, EDN1, FOXO3, MAFB, TSHZ3, RUNX2, IFNGR1, ZNF236, LAT2, PAPOLA, EHD1, SERTAD2, PELI1, KBTBD2, HNRNPA3P1, SLC39A10, KRAS, CAMTA1, NAMPT, CREBRF, ETS1, TLE4, FAR1, EDEM3, TWF1, C3orf58, SLC25A40, PSMG1, IKBIP, LCLAT1, VEZF1, SACM1L, DCAF7, ERMP1, KRT80, FLI1, DOCK4, CYR61, ICAM1, SELE, SMAD2, MYB, SKI, WTH3DI, RNF123, CSF1R, SOX6, CKAP5, JUN, PKIA, CSNK1A1, GCSAM, KDM3A, UQCRFS1, IL13RA1, FADD, BCL6, MITF, MAP3K10, NOS3, UBR4, PAXX, MECR, LUC7L2, TCEA1, ANAPC16, C17orf80, SLC27A2, XPC, EIF3G, EIF2B5, UBA3, EIF3E, PCYOX1, EXOC7, QPCTL, DYNC2H1, GLIPR2, DNAAF5, UGT8, HSPB11, CCDC137, GLB1, LRRC40, VBP1, MGST2, GNB4, JUP, DNAJC2, GOLPH3, DRAP1, NOB1, SLC1A5, RAB14, YBX3, SLC39A14, UBE2J2, EIF4A1, CAB39L, MYLK, EIF2B2, CNDP2, RTN3, TMBIM6, EIF4G2, CDC42BPB, MYD88, ADH5, EIF3C, QRICH1, CYFIP1, PSIP1, SIN3A, STK24, AGL, METAP2, SPIN1, FAM98B, ERI1, COLGALT1, UGDH, LNPK, XPO1, PALLD, MUT, RAP1B, BAG5, LPGAT1, GCFC2, EXOSC2, LNX2, ZNF248, CHD9, MEF2A, CAB39, CLUAP1, CARD11, PCDH9, ZNF561, CARHSP1, C16orf62, LIN7C, CBR4, ECI1, OSBPL10, EIF4E2, TGM2, SLC9A3R2, CHAF1B, PPFIBP1, UBXN1, ESRRA, GAPVD1, MIA2, RBM42, MFF, NEU1, SCD, UBE2D2, OVCA2, EIF3CL, AURKA, P3H3, FUBP1, RETSAT, MPP2, KIAA0368, NES, KIF22, PACSIN2, SLC25A19, IPO8, GPT2, OGFOD1, AKR7A2, TRMT1, MCAM, INA, SNX6, PDAP1, PCNT, FIP1L1, MTAP, CEP55, AIFM1, PPM1G, PAM16, PLK1, AURKB, NASP, NUPL2, NCKAP1, EXOC2, SEC24B, RRM2, GRPEL1, ALDH9A1, AP1G1, LUZP1, RPRD1A, NAA25, RP2, NUP155, OTULIN, TMX3, ERGIC1, NFYC, UBE2D3, SUZ12, GLIPR1, GPM6B, LRIF1, TAF5L, HERC4, MORC3, MBNL3, UPF2, KRT6B, FLNA, DAG1, TIMM13, BUD31, CUTA, STIM1, EOGT, GEMIN5, GNPNAT1, STXBP2, PCCB, EIF3J, CHRAC1, PLAUR, CISD2, BCL7C, TICAM2, KDM1A, PSEN1, STRN, SMARCD2, RARS, RBPJ, LARS, ALDH1A2, CNOT10, UBE2H, TNKS1BP1, PUS7, RPL39, CTSA, MBLAC2, GAR1, RAD1, NOLC1, FGF2, KDELC2, HSPA4L, PEBP1, PNPLA4, GHITM, FAM3C, PGRMC2, RPAP1, NCAPG, PDE12, TFCP2, NKX3-1, L2HGDH, PRKAR2A, ARL5A, CD109, TOMM34, CFL2, AIMP1, MIDN, CDC73, TRIO, PAXBP1, TSPAN14, INTS6, YWHAZ, PRKAR1A, SSX2IP, FAM199X, RAC1, PLS1, SAP30L, MRPS27, TNFAIP2, JUNB, SPCS1, CTNNA1, ASNS, H2AFY, NR3C1, AXL, RING1, CLTA, COL4A2, CNNM3, CTNNBL1, CNOT9, ATL2, CDK5, PPP2R2A, THOC7, DNMT1, MRPL16, RAB6C, HAX1, ABHD16A, ASB6, GPAM, HDHD5, WDR11, NMD3, IGF2R, ACOT7, RRAGA, INTS7, YARS, FLNB, CORO1B, SUPT5H, KANK2, DIAPH3, FASTKD1, NSA2, FMNL3, CHTOP, STRBP, MARCKS, POLR1B, FNDC3B, CD3EAP, PSAT1, COPS3, DEGS1, OXCT1, PDK1, EIF3A, GMPS, OLR1, SMAD4, CD68, FLT1, CEP41, CIAPIN1, CCDC82, ACTR2, TRAK1, CYP2U1, SLC35F2, ZNF493, HAL, IL17RB, TBC1D14, ZNF254, GABARAPL1, JCHAIN, RAPGEF2, WBP1L, PBRM1, ABCC4, SPECC1, MAT2B, TFPI, EXOC3, PFDN4, HK2, TYSND1, C12orf10, CRAT, PLEKHA5, PACSIN3, F5, SMARCE1, CD81, VAV2, SLC7A1, OBSCN, MAVS, DMD, CARS2, SLC12A4, MRPS34, B4GALT1, KDELC1, PDCD10, NCAPD2, UBA2, ALDH5A1, FUBP3, MYO6, NAA50, MARC2, RIOK2, OSBPL9, DDX10, ATXN10, RAB30, DEK, PHF6, ARL8B, LEMD3, ZNF207, CSE1L, CPT1A, TTC37, MAN1A2, RICTOR, IMPAD1, VPS4B, CLINT1, UBQLN2, RIF1, PNPT1, MRPL18, MAP3K14, ARMC2, LCORL, APAF1, MPP5, RAB11FIP2, NOVA1, RBAK, ARL15, MYO1D, LRRC59, CMSS1, LONP2, MUS81, ITGB4, DDB2, CAT, ATPAF1, TPBG, INTS4, TIMM8A, RBM22, MTFMT, WRB, DDRGK1, GMPPA, EEF1A2, RDH13, CLTC, NOTCH2, CARS, PTMS, CSNK1A1L, LUC7L3, TJP1, FDFT1, CDKN2A, AKR1C3, PNPLA8, S100A11, IER3IP1, FSTL1, SLC38A5, ATP6V1H, ITGB5, SRSF2, SLC7A11, ANXA2, DDX17, RAB2A, FOXK1, TMOD3, PSME4, ZNF384, GNAS, SNTB2, TMTC3, SLC30A7, ASPH, INTS8, CPD, EGFR, SRPK2, STAG2, IRF2BP2, TPRKB, VPS36, VCAM1, SMAD3, TTF1, FAM91A1, CEP83, MARF1, CDC40, DCUN1D2, KLHL5, AGO4, HBP1, WWC1, WEE1, GOLT1B, PALD1, THBS1, DBN1, DHCR24, HSD17B7, NSUN5, TPP2, UAP1, OXNAD1, SSSCA1, EEF1E1, PHGDH, CCND1, ALDH3A2, CDK2, SGPL1, TSPAN3, ANPEP, PSME3, AGRN, GNL3L, VANGL1, RTFDC1, WNT5A, TMEM167A, CLIC4, MEST, TRIM24, CDH6, MMS22L, SKIV2L2, CCR9, DR1, RSF1, ANTXR1, SEPT11, HNRNPA3, CXCL8, ZNF83, PHF14, TBC1D8B, INPP5F, ARPC3, KRCC1, FAM177A1, UBTD2, SECISBP2, PAK2, SLC33A1, ZNF28, MCM8, SMARCA4, TCF12, TOMM20, UBQLN1, PDPR, MOV10, FAM120A, FAM96B, FKBP3, STAT3, NEUROG1, EPRS, CDH2, CDH13, ATP13A1, EIF3F, NUP62, GCLC, LSM3, HTRA1, CDK4, EPB41L2, COG2, CCT2, NUCKS1, PRSS21, INTS10, STX5, CIAO1, GLG1, TROVE2, ARGLU1, CALU, BRI3BP, RAD23B, PYGL, MRS2, KIF14, POLR2C, TPD52, MTHFD2, RNF2, LTN1, TMEM33, TNPO1, GSK3B, ADAM10, ERBIN, CCL2, NFKB1, IL6, CD36, RREB1, ZKSCAN5, ZNF611, ZNF273, PDCD4, VPS18, NSD3, MASTL, MYBL1, GATM, E2F2, FAM135A, C3orf18, ARL6IP5, SHANK2, SH3PXD2A, PIK3R1, VHL, PATJ, MMP16, FOS, MAPK14, RAPH1, RAB3B, KLF9, MYC, ITK, IL2, SEL1L, DOCK1, RAD51, THRB, TERF1, ZBTB18, ZNF431, ZBTB38, ZSWIM6, ADD3, S1PR1, CBL, AGTRAP, WNK1, KCTD3, KLHL42, SP1, CHURC1, MEX3C, TWSG1, ZNF492, CCNT2, PIK3CA, DMTF1, CHD7, DCAF10, DDX3Y, CSNK1G2, ZNF714, TADA2B, UBXN2B, ACOX1, ELK4, KCTD5, ANKRD12, HIF1A, ZNF148, NAA16, ETNK2, TRPS1, PEA15, PTN, MXI1, SOCS3, ZNF98, ZNF468, ZNF300, ZNF260, ZNF160, ZKSCAN1, YEATS2, XPNPEP1, WDR82, VCPIP1, USP8, UBE2G1, TTC8, NEMP1, TMEM123, TCF4, TAPT1, STRN3, SSU72, SSH2, SLC11A2, SIRT1, SRSF1, KMT5A, SERGEF, RPS20, RGL1, REV1, PTAR1, OSTM1, NFAT5, N4BP1, MKLN1, KPNA5, KIF3A, KCNN3, INPP5A, IFIT5, HMGCS1, HLA-DPA1, GANAB, GALC, SARAF, FGL2, FEZ2, EZH1, ENTPD1, DPY19L1, CREB3L2, CPEB4, CDC37, SWSAP1, BTBD1, AKAP10, ABI2, AAK1, CHD8, AKT1, PHACTR2, XPR1, RAB3IP, KIAA1841, PCCA, RHEBP1, UQCRB, FCAMR, SLC35A1, HHIP, SELENOT, CSRP2, CDKN1B, ZFP36, KANSL1, FITM2, CYP1A1, DENND1B, FAM76A, DOK2, ZNF703, SPRED1, TMEM136, CNOT6, ADAMTS4, MTRNR2L7, MTRNR2L5, PLEKHA2, RORA, ZNF678, UBL3, PRRC1, KLHL28, EEF2, ZNF644, MTRNR2L3, CDV3, MTRNR2L9, CHRDL1, ZNF500, TDRD6, MARVELD1, CNPPD1, FOXE1, TYRP1, MAPK13, SOCS6, RPTOR, TFAM, STAT1, CCND2, CASP3, TBRG1, HOMEZ, NR1H3, PTEN, FBXW7, SAMHD1, CS, GEN1, MTRNR2L1 |
| miR-497-5p | RAF1, RUNX2, IGF1R, MAP2K1, EEF1A1, TCEA1, HIST1H3H, ALDH9A1, DESI2, HIST2H3A, PANK2, ATXN7L3B, BRD1, WEE1, DICER1, HDGF, BCL2, EIF4E, BIRC5, WNT7A, SMURF1, CHEK1, SHOC2, PLEKHA1, CEP55, ZDHHC16, CCND1, C1orf21, AMOTL1, CCND2, TARBP2, MTFR1L, USP15, FCF1, ARIH1, PAGR1, SNTB2, VPS4A, C16orf72, GOSR1, RPS6KB1, NAPG, GALNT1, CCNE1, PNPLA6, ZCCHC3, SOWAHC, UBR3, ZNRF3, YWHAH, B3GNT2, ACTR2, RARB, PI4K2B, CANX, E2F3, PPP1R11, HSPA1B, PIM1, VEGFA, CD2AP, CALU, UBN2, EN2, MAFK, FOXK1, USP42, DMTF1, ZFHX4, RAD23B, ZBTB34, RECK, ZNF275, OGT, LUZP1, CHAC1, SCAMP4, PAK2, ENTPD1, SKI, SPRED1, SMAD3, DCTN5, TNFSF9, RASSF2, ATP5G3, TNRC6B, GRAMD2B, CREBRF, PSAT1, PAFAH1B2, RPRD2, PNRC2, CDADC1, AGO4, GPR180, PPM1A, SLC39A9, FAM103A1, RBBP6, TAOK1, SNRPB2, HSPE1-MOB4, MOB4, BZW1, NUP50, FGF2, HSPA4L, RBPJ, TBPL1, FZD6, BAG4, ZNF449, YIPF6, MSL1, ELK4, GABARAPL1, ZNF691, DYNLL2, SRPRB, CDC42SE2, WIPI2, TBRG1, C1orf226, SETD1B, HOXC8, RAB3IP, PPP2R5C, SLCO3A1, CBX2, ZFP28, CHMP4B, GABPA, STRADB, MTMR3, CRIM1, SOCS5, CTDSPL, ITGA2, PIK3R1, PPIL1, CAPZA2, ZNRF2, OCRL, CHIC1, CRKL, BTRC, CDK4, CCND3, CDC25A, SIDT2, ASCC1, DMRT2, TLL1, LUC7L3, ALDH3B1, EFTUD2, CSNK1E, TPM2, ZNF460, FGFR4, DOCK11, ACTR3B, ZNF367, UBE2V1, UBE2Q1, TSC22D2, TOB2, TMEM189-UBE2V1, TMEM189, TM4SF1, TFAP2A, STK38, SSRP1, SIK1, SH3BP4, SEC24A, RNF168, REL, PISD, PDE4D, NR6A1, NFIC, NAA25, LAMC1, RUBCN, IVNS1ABP, IPPK, HOXA3, HEYL, GNB1, GNAL, FURIN, EIF1AX, DYRK3, CPSF7, CDK6, CARD10, AVL9, AKT3, AGO2, ABL2, ABHD2, ABCC6, PRKAR2A, VSIR, PHYHIP, CASKIN1, ZNRF1, CD180, KIAA0895, ORC4, ODF2L, DNAJA1, L2HGDH, ZNF622, ZMAT3, USP53, SYPL1, SRPRA, SREK1, SMAD7, PRICKLE2, LRIG2, KIF5B, FAM122B, E2F7, DDX3X, CDCA4, CDC37L1, ATG9A, ASGR2, ZNF620, HAUS3, YTHDC1, TMEM245, TMEM100, SRSF1, SNX16, SESTD1, RIMS3, RCAN3, PPIG, PLRG1, PLAG1, PHKA1, MYO5A, KIF23, HNRNPDL, CDK1, CCNE2, CBX6, AXIN2, CASK, DMPK, ATAD5, AKR1B10, GPATCH8, ARHGDIA, CPEB3, JARID2, CAMSAP1, ATG14, TRIM35, FLCN, NNT, SLC2A3, SBNO1, POM121C, NUFIP2, LAMP2, EFNB2, RPL14, GNAT1, HOXA10, CBX4, PHC3, PDCD1, BAZ2A, APP, ZNF585B, ANAPC13, PRSS21, RALGAPB, GSG1, POLDIP3, AP5Z1, CLSPN, UGT2B4, MAP4K2, CCDC83, DECR1, ZBTB10, YWHAQ, USP48, SALL1, RUNX1T1, RTN4, PNISR, PHLPP2, PAG1, NUCKS1, MKX, MBD4, LRRFIP2, KPNA3, KPNA1, HIGD1A, HCFC2, GRB2, FKBP1A, FBXL20, FASN, CRK, CLIP4, CDK17, CACUL1, C11orf24, ASH1L, AMOT, SLC29A1, OSCAR, MTHFR, FAM229B, EPM2AIP1, ZNF267, SSU72, DNAJC10, ZNF704, YRDC, TPM3, TMEM161B, TM7SF3, TAF13, SZRD1, RNF149, RACGAP1, RAB23, PTPRD, PRKAA1, PRDM4, PLPP3, LURAP1L, KANK1, HIST2H2BE, GPR27, EXT1, CYP26B1, CREBL2, CNKSR3, CA8, BTN3A3, ARHGAP12, OSBPL3, KRT33B, TUBB2A, MSANTD4, LANCL1, HNRNPA2B1, KIAA1456, SLC25A12, DLGAP3, THRAP3, SMDT1, RAPH1, CCNT1, ZNF391, CCDC80, ZBTB16, XKR7, WNK3, VAV2, TGFBR3, RASEF, NCKAP1, MAP3K7, KLHL15, GNG12, FZD9, CMTM4, CCDC88C, ARMC12, AHNAK2, ACVR2A, TLK1, UBE2H, TTLL5, RIF1, SERBP1, PHF19, LSM11, PLPBP, CLEC2D, PDIA6, N4BP1, TRAK1, ADRA2B, ANKMY1, GPRC5A, C3orf36, BSPRY, ANKRD36, KLHL40, NOTCH2, EIF2B2, CUL3, DCAF17, RS1, GLP2R, FLOT2, HNRNPA1L2, TPM1, NEGR1, MCFD2, HNRNPA1, SLC35E2B, ARHGAP32, RAB15, ADORA3, PPIP5K2, SYNRG, KCNN4, ANLN, Reck, MACC1, PBX3, MTOR, TWIST1, IKBKB, ESRRA, AP2B1, AURKAIP1, BCL2L12, C16orf58, CLU, CLUH, DENND6A, DIAPH1, FBXL18, GATAD2A, HSPA8, IER2, KMT2D, MAP2K3, PLEKHB2, POLR2E, PPP6R3, RNPS1, RPRD1B, SEC61A1, SNCG, WDR13, C21orf62, CARM1, CD274, JPT2, MINK1, RFK, TXNIP, VOPP1, ZNF284 |
| miR-199a-5p | EZH2, IKBKB, CCNL1, LIF, JUNB, MED6, MECP2, ETS2, DDR1, EDN1, MAP3K11, HIF1A, SOX9, SMARCA2, CD44, TMEM54, SMAD4, SULT1E1, GPR78, ERBB2, UNG, CAV1, SIRT1, HSPA5, ATF6, ERN1, KL, APOE, DNAJA4, ERBB3, CDH1, PTGS2, RNF11, ZNF544, TFDP2, ZNF844, PANK3, COL19A1, LIN7A, ARHGAP12, CTSC, RND1, NECTIN1, DRAM1, BECN1, MAFB, WNK1, NFKB1, ACVR1B, VEGFA, CDH2, SNAI1, GSK3B, FZD4, WNT2, JAG1, PSG11, PSG3, PIN1, C1orf226, PLEKHG2, PDE11A, SNTB1, SLC38A2, SESN2, RER1, PLXND1, MAP3K9, E2F3, COX15, TSC22D1, CEP120, SLC16A10, POLR2F, RCC1, DYNAP, PAX8, EXTL3, ZNF669, ZNF440, ZNF117, ZNF791, ZNF772, ZNF394, TUBG1, NAA15, DDX19B, RIC8A, C3orf36, SNRNP48, PSAPL1, AKAP17A, PLGRKT, TNFRSF13C, SLC8A1, CSGALNACT1, SERPINH1, TMOD2, DDX3X, ZNF525, ZNF195, ZNF415, ZNF468, ZNF611, ZNF215, ZNF286B, ZFP1, ZNF846, ZNF625, ZNF584, TRIM10, VAV3, OXSR1, PLPP4, A2ML1, RNF115, AGTRAP, NDUFS2, CHRFAM7A, CRIPT, POLA2, GATA6, CDK9, VPS53, PTCD2, SLC26A2, PODXL, ABCC1, TBC1D21, CENPO, CHCHD4, LAX1, SNAP25, HK2, KRAS, SMAD3, ETS1, CCR7, PDE4D, CTGF, TGFB2, PIK3CD, SETD2, LDLR, CLTC, RAB21, OSCP1, PIAS3, PSMD9, CDKN1C, ITGA3, FZD6, MAP4K3, TGFBR1, SLC27A1, C16orf58, CSNK2A1, DDI2, GM2A, NAB2, VASP, ZBTB37, QSOX1, ZDHHC9, DDHD1, XRRA1 |
