# Supplementary material for: Extracellular Vesicles Mediate Mesenchymal Stromal Cell-Dependent Regulation of B Cell PI3K-AKT Signaling Pathway and Actin Cytoskeleton
Source: Front Immunol. 2019 Mar 12;10:446. doi: 10.3389/fimmu.2019.00446 (PMC6423067; doi:10.3389/fimmu.2019.00446)
Supplement: Supplementary file 6 [file Table_6.DOCX]

**Supplementary information, Table S6.** Pathway enrichment analysis on miR-155-5p, miR-497-5p and miR-199a-5p experimentally validated targets (top 20 KEGG pathways are shown).

| hsa-miR-155-5p |  |  |
| --- | --- | --- |
| Term | Gene number (%) | adj p-value |
| Pathways in cancer  PI3K-Akt signaling pathway  HTLV-I infection  Hepatitis B  FoxO signaling pathway  Proteoglycans in cancer  Focal adhesion  Signaling pathways regulating pluripotency of stem cells  TNF signaling pathway  RNA transport  Influenza A  Hepatitis C  Transcriptional misregulation in cancer  Colorectal cancer  T cell receptor signaling pathway  Measles  Chagas disease (American trypanosomiasis)  Osteoclast differentiation  AMPK signaling pathway  Ubiquitin mediated proteolysis | 5,8  4,1  4  3,2  2,7  2,7  2,7  2,5  2,4  2,4  2,4  2,3  2,3  2,2  2,2  2,2  2,1  2,1  2  2 | 1,10E-05  1,10E-02  1,90E-04  4,30E-06  1,80E-04  2,00E-02  2,50E-02  7,80E-04  6,00E-05  1,70E-02  1,80E-02  2,60E-03  2,30E-02  5,20E-07  3,20E-04  6,50E-03  1,00E-03  1,10E-02  1,20E-02  2,80E-02 |
| hsa-miR-497-5p |  |  |
| Term | Gene number (%) | adj p-value |
| Pathways in cancer  PI3K-Akt signaling pathway  MicroRNAs in cancer  MAPK signaling pathway  Viral carcinogenesis  Focal adhesion  Cell cycle  Insulin signaling pathway  Signaling pathways regulating pluripotency of stem cells  Hepatitis B  Proteoglycans in cancer  HTLV-I infection  Epstein-Barr virus infection  Ras signaling pathway  Hippo signaling pathway  Small cell lung cancer  Prostate cancer  FoxO signaling pathway  Acute myeloid leukemia  Chronic myeloid leukemia | 7,2  6,5  4,8  4,1  3,9  3,9  3,7  3,5  3,5  3,5  3,5  3,5  3,3  3,3  3  2,8  2,8  2,8  2,6  2,6 | 1,60E-05  1,80E-05  1,20E-03  4,10E-03  1,30E-03  1,30E-03  2,50E-05  2,50E-04  2,70E-04  3,40E-04  5,90E-03  3,90E-02  9,30E-03  3,50E-02  4,00E-03  2,20E-04  2,30E-04  4,50E-03  3,10E-05  2,10E-04 |
| hsa-miR-199a-5p |  |  |
| Term | Gene number (%) | adj p-value |
| Pathways in cancer  HTLV-I infection  MicroRNAs in cancer  Proteoglycans in cancer  Pancreatic cancer  Signaling pathways regulating pluripotency of stem cells  Hippo signaling pathway  Hepatitis B  Adherens junction  Chronic myeloid leukemia  FoxO signaling pathway  Colorectal cancer  Prostate cancer  HIF-1 signaling pathway  TNF signaling pathway  Cell cycle  Non-alcoholic fatty liver disease (NAFLD)  B cell receptor signaling pathway  Small cell lung cancer  Melanogenesis | 12  9,6  7,8  7,2  6,6  6  6  5,4  4,8  4,8  4,8  4,2  4,2  4,2  4,2  4,2  4,2  3,6  3,6  3,6 | 7,40E-06  1,20E-05  1,90E-03  5,50E-04  5,20E-07  8,60E-04  1,40E-03  4,40E-03  7,60E-04  6,60E-04  1,30E-02  1,50E-03  8,00E-03  1,20E-02  1,50E-02  2,80E-02  5,60E-02  1,40E-02  3,00E-02  5,10E-02 |
