# Supplementary material for: Extracellular Vesicles Mediate Mesenchymal Stromal Cell-Dependent Regulation of B Cell PI3K-AKT Signaling Pathway and Actin Cytoskeleton
Source: Front Immunol. 2019 Mar 12;10:446. doi: 10.3389/fimmu.2019.00446 (PMC6423067; doi:10.3389/fimmu.2019.00446)
Supplement: Supplementary file 7 [file Table_7.DOCX]

**Supplementary Information, Table S7.** Loadings on PC1 of the first 200 variables included by Ranking-PCA merging miRNA and proteomic data for both EVs and MSCs. Variables are listed in order of inclusion by Ranking-PCA.

| EVs | | | MSCs | | |
| --- | --- | --- | --- | --- | --- |
| Order of inclusion  by Ranking-PCA | **Variable** | **loadings on PC1** | **Order of inclusion**  **by Ranking-PCA** | **Variable** | **loadings on PC1** |
| 1 | hsa-miR-193b-3p | 0.078085 | 1 | hsa-miR-146b-3p | 0.074598 |
| 2 | hsa-miR-142-5p | 0.065747 | 2 | hsa-miR-143-5p | -0.07444 |
| 3 | hsa-miR-21-5p | -0.07851 | 3 | hsa-miR-28-5p | 0.074518 |
| 4 | hsa-miR-222-3p | -0.07351 | 4 | hsa-miR-26b-3p | -0.07445 |
| 5 | CALM | -0.07058 | 5 | hsa-miR-328-3p | -0.07424 |
| 6 | TPM2 | -0.07971 | 6 | hsa-miR-148a-5p | -0.07403 |
| 7 | hsa-miR-224-5p | -0.06595 | 7 | hsa-miR-146b-5p | 0.073847 |
| 8 | hsa-miR-143-3p | -0.0804 | 8 | hsa-miR-125b-2-3p | -0.07396 |
| 9 | hsa-miR-185-5p | -0.05751 | 9 | hsa-let-7f-5p | 0.074328 |
| 10 | VIME | -0.08363 | 10 | hsa-miR-424-3p | 0.073985 |
| 11 | hsa-miR-629-5p | 0.055107 | 11 | hsa-miR-874-3p | -0.07424 |
| 12 | hsa-miR-126-3p | 0.082491 | 12 | hsa-miR-382-5p | 0.074017 |
| 13 | hsa-miR-221-5p | -0.06161 | 13 | hsa-let-7i-5p | 0.074116 |
| 14 | hsa-miR-132-3p | 0.066751 | 14 | hsa-miR-1276 | -0.07221 |
| 15 | hsa-miR-485-5p | -0.05812 | 15 | hsa-miR-32-5p | 0.073731 |
| 16 | MOES | -0.07947 | 16 | hsa-miR-149-5p | -0.07415 |
| 17 | hsa-miR-199a-5p | -0.05395 | 17 | hsa-miR-28-3p | 0.073542 |
| 18 | hsa-let-7a-5p | -0.07549 | 18 | hsa-let-7a-5p | 0.073508 |
| 19 | MED6 | 0.080234 | 19 | hsa-miR-155-5p | 0.073754 |
| 20 | hsa-miR-23b-3p | 0.048775 | 20 | hsa-miR-92a-3p | 0.07238 |
| 21 | hsa-miR-196a-5p | -0.08271 | 21 | hsa-miR-99a-5p | 0.073624 |
| 22 | hsa-miR-146b-5p | 0.052877 | 22 | hsa-let-7b-3p | -0.07447 |
| 23 | hsa-let-7i-5p | -0.07156 | 23 | hsa-miR-574-3p | 0.074337 |
| 24 | APOB | 0.063557 | 24 | hsa-miR-3909 | -0.07419 |
| 25 | hsa-miR-125b-5p | -0.06961 | 25 | hsa-miR-1296-5p | -0.0737 |
| 26 | A2MG | 0.060184 | 26 | hsa-miR-7-5p | 0.074119 |
| 27 | hsa-miR-31-3p | 0.066542 | 27 | hsa-miR-196a-5p | 0.07339 |
| 28 | hsa-miR-16-2-3p | -0.05081 | 28 | hsa-miR-1287-5p | -0.07365 |
| 29 | CO4A | 0.079064 | 29 | hsa-miR-30e-3p | -0.07272 |
| 30 | hsa-miR-125b-2-3p | -0.05098 | 30 | hsa-miR-1343-3p | -0.07158 |
| 31 | ANXA6 | -0.07553 | 31 | hsa-miR-3199 | 0.072956 |
| 32 | hsa-miR-9-5p | 0.053276 | 32 | hsa-miR-221-3p | -0.07363 |
| 33 | hsa-miR-25-3p | 0.083516 | 33 | hsa-miR-125a-5p | 0.073971 |
| 34 | hsa-miR-361-3p | 0.046741 | 34 | hsa-let-7d-5p | 0.071781 |
| 35 | ITIH3 | 0.084856 | 35 | hsa-miR-218-5p | 0.072665 |
| 36 | hsa-miR-485-3p | -0.0424 | 36 | hsa-miR-186-5p | 0.074092 |
| 37 | APMAP | 0.078659 | 37 | hsa-miR-145-5p | -0.07347 |
| 38 | hsa-miR-181a-2-3p | -0.05683 | 38 | hsa-miR-671-3p | -0.07402 |
| 39 | hsa-miR-425-5p | 0.070717 | 39 | hsa-miR-34a-3p | 0.072359 |
| 40 | hsa-miR-16-5p | 0.045908 | 40 | hsa-miR-502-5p | -0.07268 |
| 41 | RAB1A | -0.07595 | 41 | hsa-miR-548ay-3p | 0.073777 |
| 42 | hsa-miR-1343-3p | 0.045342 | 42 | hsa-miR-181c-3p | -0.07335 |
| 43 | LG3BP | 0.082233 | 43 | hsa-miR-363-3p | 0.072762 |
| 44 | hsa-miR-148a-5p | 0.044039 | 44 | hsa-miR-4326 | -0.07333 |
| 45 | hsa-miR-100-5p | -0.08212 | 45 | hsa-let-7g-5p | 0.073696 |
| 46 | hsa-miR-589-5p | -0.04105 | 46 | hsa-miR-26a-5p | 0.073384 |
| 47 | THBG | 0.081678 | 47 | hsa-miR-323a-3p | 0.073754 |
| 48 | hsa-miR-30e-3p | 0.037992 | 48 | hsa-miR-484 | 0.071501 |
| 49 | PTX3 | 0.082514 | 49 | hsa-miR-24-3p | 0.072904 |
| 50 | hsa-miR-382-5p | 0.037431 | 50 | hsa-miR-671-5p | -0.0719 |
| 51 | A1AT | 0.076496 | 51 | hsa-miR-34c-3p | 0.0724 |
| 52 | hsa-miR-204-5p | 0.042234 | 52 | hsa-miR-30c-2-3p | -0.07307 |
| 53 | AAAT | -0.08098 | 53 | hsa-miR-23a-3p | -0.07337 |
| 54 | hsa-miR-192-5p | 0.041076 | 54 | hsa-miR-455-5p | 0.072971 |
| 55 | TSP1 | -0.07379 | 55 | hsa-miR-3200-3p | -0.0713 |
| 56 | hsa-miR-200c-3p | -0.0481 | 56 | hsa-miR-3187-3p | 0.073098 |
| 57 | hsa-miR-502-3p | 0.060431 | 57 | hsa-miR-29a-3p | 0.0738 |
| 58 | LUM | 0.064723 | 58 | TCPA | 0.072468 |
| 59 | hsa-miR-221-3p | 0.059068 | 59 | hsa-miR-199a-5p | -0.07334 |
| 60 | hsa-let-7a-3p | -0.06378 | 60 | hsa-miR-3614-3p | 0.07296 |
| 61 | hsa-miR-140-3p | 0.05944 | 61 | hsa-miR-134-5p | 0.071696 |
| 62 | hsa-miR-145-3p | -0.04764 | 62 | hsa-miR-362-5p | -0.07014 |
| 63 | ICAM1 | 0.066196 | 63 | hsa-miR-30c-1-3p | -0.07251 |
| 64 | H2B2F | 0.054812 | 64 | hsa-miR-30b-5p | 0.071202 |
| 65 | KV201 | 0.057587 | 65 | hsa-miR-197-3p | -0.07301 |
| 66 | hsa-let-7e-5p | -0.0635 | 66 | hsa-miR-128-3p | -0.07272 |
| 67 | hsa-miR-29b-3p | 0.046879 | 67 | hsa-miR-625-3p | -0.07012 |
| 68 | hsa-miR-380-5p | 0.063632 | 68 | hsa-miR-10a-5p | 0.072272 |
| 69 | hsa-miR-4755-3p | 0.057817 | 69 | hsa-miR-218-1-3p | 0.071971 |
| 70 | FBLN1 | 0.064434 | 70 | hsa-miR-452-3p | -0.07222 |
| 71 | hsa-miR-615-3p | -0.04226 | 71 | hsa-miR-1271-5p | 0.07345 |
| 72 | ANXA4 | -0.08113 | 72 | hsa-miR-127-5p | -0.07313 |
| 73 | hsa-miR-26a-5p | -0.03317 | 73 | hsa-miR-708-3p | 0.072372 |
| 74 | ANXA2 | -0.08327 | 74 | hsa-miR-6827-5p | -0.07028 |
| 75 | hsa-miR-409-5p | 0.033702 | 75 | hsa-miR-222-3p | -0.07339 |
| 76 | hsa-miR-142-3p | 0.059944 | 76 | hsa-miR-505-3p | -0.07205 |
| 77 | hsa-miR-107 | 0.060962 | 77 | hsa-miR-98-5p | 0.070355 |
| 78 | hsa-miR-140-5p | -0.04365 | 78 | hsa-let-7a-3p | -0.07301 |
| 79 | 1433B | -0.0828 | 79 | hsa-miR-660-3p | -0.07251 |
| 80 | hsa-let-7b-5p | -0.04781 | 80 | hsa-miR-452-5p | -0.0706 |
| 81 | CO3 | 0.036096 | 81 | hsa-miR-127-3p | 0.070952 |
| 82 | hsa-miR-27a-5p | 0.070649 | 82 | hsa-miR-1249-5p | 0.06825 |
| 83 | hsa-miR-483-3p | 0.031431 | 83 | hsa-miR-629-5p | -0.07129 |
| 84 | COF1 | -0.07988 | 84 | hsa-miR-181b-5p | 0.072575 |
| 85 | hsa-miR-299-3p | 0.028535 | 85 | CATB | 0.063694 |
| 86 | ITB1 | -0.08353 | 86 | hsa-miR-485-3p | -0.07189 |
| 87 | hsa-miR-769-5p | -0.04903 | 87 | hsa-miR-1-3p | -0.07206 |
| 88 | hsa-miR-340-3p | 0.028528 | 88 | hsa-let-7e-3p | -0.07145 |
| 89 | 5NTD | -0.08376 | 89 | hsa-let-7c-5p | 0.072683 |
| 90 | hsa-miR-20a-5p | 0.026674 | 90 | hsa-miR-379-3p | -0.07135 |
| 91 | CDC42 | -0.07917 | 91 | hsa-miR-107 | -0.0719 |
| 92 | hsa-let-7b-3p | -0.0255 | 92 | hsa-miR-92b-5p | 0.072531 |
| 93 | GBG12 | -0.08201 | 93 | hsa-miR-181a-2-3p | -0.07301 |
| 94 | hsa-miR-223-5p | -0.02533 | 94 | hsa-miR-369-5p | -0.07038 |
| 95 | MVP | -0.08285 | 95 | hsa-miR-378a-5p | -0.07162 |
| 96 | hsa-miR-29a-3p | 0.0443 | 96 | hsa-miR-500a-3p | -0.07152 |
| 97 | hsa-miR-99a-5p | -0.02774 | 97 | hsa-miR-4636 | -0.07036 |
| 98 | hsa-miR-152-5p | 0.05737 | 98 | hsa-miR-181a-3p | -0.07204 |
| 99 | ITIH2 | 0.081485 | 99 | hsa-miR-221-5p | -0.0729 |
| 100 | hsa-miR-625-3p | -0.02442 | 100 | hsa-miR-4645-3p | 0.071432 |
| 101 | hsa-miR-106b-3p | 0.055012 | 101 | hsa-miR-23b-3p | -0.07262 |
| 102 | PEDF | 0.06092 | 102 | hsa-miR-224-5p | -0.07194 |
| 103 | hsa-miR-215-5p | 0.058927 | 103 | MARCS | 0.06645 |
| 104 | hsa-miR-6716-3p | -0.03173 | 104 | hsa-miR-31-5p | -0.07079 |
| 105 | COCA1 | -0.07785 | 105 | hsa-miR-30a-3p | -0.06964 |
| 106 | hsa-miR-450b-5p | 0.02955 | 106 | hsa-miR-101-3p | 0.069179 |
| 107 | IF4E | 0.067213 | 107 | hsa-miR-1185-5p | -0.06711 |
| 108 | hsa-miR-377-3p | 0.047607 | 108 | hsa-miR-4637 | 0.071066 |
| 109 | VTNC | 0.062282 | 109 | hsa-miR-654-3p | -0.07246 |
| 110 | hsa-miR-212-5p | -0.04674 | 110 | hsa-miR-483-3p | -0.07013 |
| 111 | LEG1 | -0.0838 | 111 | hsa-miR-148b-5p | 0.07197 |
| 112 | GBB1 | -0.08383 | 112 | hsa-miR-483-5p | -0.06516 |
| 113 | BASP1 | -0.08381 | 113 | hsa-miR-146a-5p | 0.070831 |
| 114 | AMPN | -0.08388 | 114 | hsa-miR-1185-2-3p | -0.07218 |
| 115 | S10A6 | -0.08355 | 115 | hsa-miR-580-3p | 0.071643 |
| 116 | S10AB | -0.08357 | 116 | hsa-miR-7705 | 0.07075 |
| 117 | hsa-miR-21-3p | 0.0831 | 117 | hsa-miR-548o-3p | -0.07222 |
| 118 | RHOA | -0.08272 | 118 | ACTB | -0.06409 |
| 119 | ANXA1 | -0.08346 | 119 | hsa-miR-449c-5p | 0.071447 |
| 120 | CSPG2 | 0.081736 | 120 | hsa-miR-99b-5p | 0.071877 |
| 121 | hsa-miR-331-5p | 0.078553 | 121 | hsa-miR-26b-5p | 0.069603 |
| 122 | TPIS | -0.08331 | 122 | hsa-miR-4683 | 0.069722 |
| 123 | SH3L3 | -0.08287 | 123 | hsa-miR-758-3p | -0.0715 |
| 124 | FLNA | -0.083 | 124 | hsa-miR-381-5p | -0.0721 |
| 125 | CD81 | -0.08284 | 125 | hsa-let-7d-3p | -0.07221 |
| 126 | PLST | -0.08293 | 126 | hsa-miR-339-3p | -0.06758 |
| 127 | TAGL | -0.08285 | 127 | hsa-miR-129-5p | 0.071975 |
| 128 | MARCS | -0.0827 | 128 | RL7A | -0.06396 |
| 129 | AHNK | -0.08255 | 129 | hsa-miR-4773 | 0.071118 |
| 130 | 1433Z | -0.08106 | 130 | hsa-miR-93-3p | -0.07137 |
| 131 | CD59 | -0.08225 | 131 | ACTBL | -0.06409 |
| 132 | ACTN1 | -0.08209 | 132 | hsa-miR-224-3p | -0.06742 |
| 133 | VINC | -0.08186 | 133 | hsa-miR-5010-3p | 0.071097 |
| 134 | EF1A1 | -0.08088 | 134 | hsa-miR-132-3p | 0.071073 |
| 135 | RAB7A | -0.08142 | 135 | hsa-miR-200c-3p | 0.067644 |
| 136 | ACTB | -0.08146 | 136 | hsa-miR-769-5p | -0.06904 |
| 137 | RTN4 | -0.08096 | 137 | hsa-miR-6881-3p | -0.07057 |
| 138 | MYOF | -0.08021 | 138 | hsa-miR-361-3p | 0.07099 |
| 139 | SERA | -0.08036 | 139 | hsa-miR-487a-5p | 0.065621 |
| 140 | LDHB | -0.07993 | 140 | hsa-miR-4668-5p | 0.065365 |
| 141 | PRDX1 | -0.07963 | 141 | hsa-miR-450a-5p | -0.06948 |
| 142 | THIO | -0.08066 | 142 | PRDX4 | -0.0634 |
| 143 | TCPD | -0.08067 | 143 | hsa-miR-29b-3p | 0.071265 |
| 144 | GRP78 | -0.08074 | 144 | hsa-miR-132-5p | 0.070999 |
| 145 | ADHX | -0.08074 | 145 | hsa-miR-216b-5p | 0.060468 |
| 146 | CD63 | -0.08106 | 146 | hsa-miR-296-3p | -0.0701 |
| 147 | MYL6 | -0.08093 | 147 | hsa-miR-433-3p | -0.07058 |
| 148 | EF2 | -0.08073 | 148 | hsa-miR-6782-3p | -0.06311 |
| 149 | CAP1 | -0.08071 | 149 | FINC | 0.069743 |
| 150 | CRYAB | -0.08069 | 150 | hsa-miR-6852-5p | 0.068383 |
| 151 | VAT1 | -0.0809 | 151 | hsa-miR-342-3p | 0.070275 |
| 152 | CPNS1 | -0.08061 | 152 | hsa-miR-1254 | -0.07036 |
| 153 | TARSH | 0.080605 | 153 | hsa-miR-3613-5p | -0.07078 |
| 154 | PPIA | -0.07923 | 154 | hsa-miR-143-3p | -0.07166 |
| 155 | PLP2 | -0.08086 | 155 | hsa-miR-345-5p | -0.07063 |
| 156 | CAV1 | -0.08017 | 156 | hsa-miR-23b-5p | -0.07003 |
| 157 | CALD1 | -0.08008 | 157 | hsa-miR-93-5p | -0.07012 |
| 158 | PGAM1 | -0.07999 | 158 | hsa-miR-494-5p | -0.06785 |
| 159 | TBB5 | -0.08139 | 159 | hsa-miR-29b-2-5p | 0.070301 |
| 160 | TRFE | 0.080264 | 160 | hsa-miR-125b-1-3p | 0.070708 |
| 161 | ARF1 | -0.07446 | 161 | hsa-miR-15a-5p | -0.06862 |
| 162 | TERA | -0.07959 | 162 | hsa-miR-337-3p | -0.07072 |
| 163 | PGK1 | -0.07977 | 163 | KCRB | -0.06705 |
| 164 | 4F2 | -0.07578 | 164 | hsa-miR-409-5p | -0.07008 |
| 165 | LOXL2 | -0.07797 | 165 | hsa-miR-616-5p | -0.0679 |
| 166 | ALDOA | -0.07897 | 166 | hsa-miR-654-5p | -0.06624 |
| 167 | HSP7C | -0.07995 | 167 | PDLI2 | -0.07031 |
| 168 | TPM4 | -0.07988 | 168 | hsa-miR-1291 | 0.064829 |
| 169 | 1433E | -0.0781 | 169 | hsa-miR-10b-5p | 0.071307 |
| 170 | hsa-miR-374a-3p | -0.0715 | 170 | hsa-miR-34b-5p | 0.067951 |
| 171 | KPYM | -0.07861 | 171 | hsa-miR-450a-2-3p | -0.06463 |
| 172 | CH60 | -0.07791 | 172 | hsa-miR-652-3p | -0.07069 |
| 173 | ANXA5 | -0.08094 | 173 | hsa-miR-579-5p | 0.063915 |
| 174 | EHD1 | -0.07016 | 174 | hsa-miR-5001-3p | 0.070064 |
| 175 | CO1A2 | -0.07783 | 175 | hsa-miR-3940-3p | -0.06185 |
| 176 | TBA4A | -0.07617 | 176 | hsa-miR-548l | -0.06867 |
| 177 | PROF1 | -0.07921 | 177 | hsa-miR-30c-5p | -0.07036 |
| 178 | CO1A1 | -0.07764 | 178 | hsa-miR-1294 | -0.06567 |
| 179 | HSPB1 | -0.07785 | 179 | hsa-miR-181d-5p | 0.069591 |
| 180 | ITIH1 | 0.077591 | 180 | hsa-miR-378a-3p | -0.06889 |
| 181 | COR1C | -0.07677 | 181 | MYO1C | -0.06565 |
| 182 | TRFL | 0.077259 | 182 | hsa-miR-192-5p | 0.068334 |
| 183 | ITAV | -0.06786 | 183 | hsa-miR-598-3p | -0.06707 |
| 184 | hsa-miR-150-5p | 0.079216 | 184 | TPIS | -0.06948 |
| 185 | CO3A1 | -0.07595 | 185 | hsa-miR-365b-5p | -0.05862 |
| 186 | G3P | -0.08085 | 186 | hsa-miR-659-5p | 0.065403 |
| 187 | ELN | -0.07564 | 187 | hsa-miR-628-5p | 0.064524 |
| 188 | ADT3 | -0.07524 | 188 | hsa-miR-4493 | 0.06731 |
| 189 | TBA1B | -0.0791 | 189 | hsa-miR-641 | -0.06511 |
| 190 | NB5R3 | -0.07465 | 190 | hsa-miR-1262 | 0.066634 |
| 191 | ARF3 | -0.07962 | 191 | hsa-miR-331-3p | -0.0684 |
| 192 | GSTP1 | -0.06433 | 192 | hsa-miR-708-5p | 0.068702 |
| 193 | MYH9 | -0.07518 | 193 | ANXA5 | -0.06968 |
| 194 | hsa-miR-299-5p | -0.07434 | 194 | hsa-miR-200b-3p | 0.06214 |
| 195 | ARP2 | -0.07432 | 195 | hsa-miR-18a-3p | 0.067722 |
| 196 | LDHA | -0.07746 | 196 | hsa-miR-324-3p | 0.063821 |
| 197 | DEST | -0.07528 | 197 | hsa-miR-154-3p | 0.068055 |
| 198 | DYHC1 | -0.07411 | 198 | hsa-miR-361-5p | -0.06907 |
| 199 | ATPA | -0.07333 | 199 | hsa-miR-299-3p | -0.06912 |
| 200 | RAP1B | -0.06893 | 200 | hsa-miR-92b-3p | -0.06902 |
