# Supplementary material for: Extracellular Vesicles Mediate Mesenchymal Stromal Cell-Dependent Regulation of B Cell PI3K-AKT Signaling Pathway and Actin Cytoskeleton
Source: Front Immunol. 2019 Mar 12;10:446. doi: 10.3389/fimmu.2019.00446 (PMC6423067; doi:10.3389/fimmu.2019.00446)
Supplement: Supplementary file 8 [file Table_8.DOCX]

**_Supplementary information, Table S8_**_. List of antibodies used for western blotting validation_

| Protein | Primary Antibody | Primary Antibody  dilution used | Secondary Antibody | Secondary Antibody  dilution used |
| --- | --- | --- | --- | --- |
| CD44 molecule | CD44  GeneTex  (GTX628472) | 1:500 | Anti-Mouse  Santa Cruz Biotechnology  (sc-516102) | 1:2000 |
| CD63 molecule | CD63  GeneTex  (GTX17441) | 1:500 | Anti-Rabbit  Santa Cruz Biotechnology  (sc-2004) | 1:5000 |
| Endoglin | CD105  GeneTex  (GTX100508) | 1:500 | Anti-Rabbit  Santa Cruz Biotechnology  (sc-2004) | 1:5000 |
| Galectin-3 binding protein | Mac-2BP  Santa Cruz Biotechnology  (sc-374541) | 1:100 | Anti-Mouse  Santa Cruz Biotechnology  (sc-516102) | 1:1000 |
| Melanoma cell adhesion molecule | CD146  GeneTex  (GTX108777) | 1:500 | Anti-Rabbit  Santa Cruz Biotechnology  (sc-2004) | 1:5000 |
| Moesin | Moesin  Cell Signaling  (#3150) | 1:1000 | Anti-Rabbit  Santa Cruz Biotechnology  (sc-2004) | 1:5000 |
| Pentatraxin3 | Ptx3  Santa Cruz Biotechnology  (sc-373951) | 1:200 | Anti-Mouse  Santa Cruz Biotechnology  (sc-516102) | 1:1000 |
| S-100A6 | Calcyclin  Santa Cruz Biotechnology  (sc-271396) | 1:200 | Anti-Mouse  Santa Cruz Biotechnology  (sc-516102) | 1:1000 |
