# Supplementary material for: Extracellular Vesicles Mediate Mesenchymal Stromal Cell-Dependent Regulation of B Cell PI3K-AKT Signaling Pathway and Actin Cytoskeleton
Source: Front Immunol. 2019 Mar 12;10:446. doi: 10.3389/fimmu.2019.00446 (PMC6423067; doi:10.3389/fimmu.2019.00446)
Supplement: Supplementary file 9 [file Data_Sheet_1.docx]

**Supplementary Materials and Methods**

**Shotgun Mass Spectrometry**

The stationary phase was a Halo C18 column (0.5 x 100 mm, 2.7 µm). The mobile phase was a mixture of 0.1% (v/v) formic acid in water (A) and 0.1% (v/v) formic acid in acetonitrile (B), eluting at a flow-rate of 15.0 µL min−1 at an increasing concentration of solvent B from 2% to 40% in 30 min. The injection volume was 4.0 μl and the oven temperature was set at 40°C. For identification purposes, the samples were subjected to a data dependent acquisition (DDA): the mass spectrometer analysis was performed using a mass range of 100–1500 Da (TOF scan with an accumulation time of 0.25 s), followed by a MS/MS product ion scan from 200 to 1250 Da (accumulation time of 5.0 ms), with the abundance threshold set at 30 cps (35 candidate ions can be monitored during every cycle). The ion source parameters in electrospray positive mode were set as follows: curtain gas (N2) at 25 psig, nebulizer gas GAS1 at 25 psig, and GAS2 at 20 psig, ionspray floating voltage (ISFV) at 5000 V, source temperature at 450 °C and declustering potential at 25 V. For the label-free quantification, samples were subjected to cyclic data independent analysis (DIA) of the mass spectra, using a 25-Da window: the mass spectrometer was operated such that a 50-ms survey scan (TOF-MS) was performed and subsequent MS/MS experiments were performed on all precursors. These MS/MS experiments were performed in a cyclic manner using an accumulation time of 40 ms per 25-Da swath (36 swaths in total) for a total cycle time of 1.5408 s. The ions were fragmented for each MS/MS experiment in the collision cell using the rolling collision energy.

**Univariate and multivariate statistical analysis**

PCA and graphical representations were carried out by Statistica v. 7.1 (StatSoft Inc., Tulsa, OK, USA). For both EVs and MSCs, proteomic data were arranged in a matrix with 42 rows (7 individuals x 2 conditions - control and primed - x 3 replications) and 215 and 668 columns, respectively (the protein counts). In both cases, the data were pre-treated as follows: centering on each individual independently, to remove biological information related to the patient, and autoscaling (global centering and normalization to unit variance) to remove scale effects between the variables. Only proteins expressed in at least 4 individuals over 7 were considered.

PCA was also performed to combine proteomic and miRNA datasets. In this case, for both EVs and MSCs, data were arranged in a matrix with 24 rows (4 individuals x 2 conditions - control and primed - x 3 replications) and 632 (424 miRNA signals and 208 proteins) and 1499 columns (837 miRNA signals + 662 proteins), respectively. In both cases, the data were pre-treated as before: centering on each individual independently, to remove biological information related to the patient, and autoscaling (global centering and normalization to unit variance) to remove scale effects between the variables. Only proteins expressed in at least 2 individuals over 4 were considered.
