# Supplementary figures and images for: Extracellular Vesicles Mediate Mesenchymal Stromal Cell-Dependent Regulation of B Cell PI3K-AKT Signaling Pathway and Actin Cytoskeleton
Source: Front Immunol. 2019 Mar 12;10:446. doi: 10.3389/fimmu.2019.00446 (PMC6423067; doi:10.3389/fimmu.2019.00446)

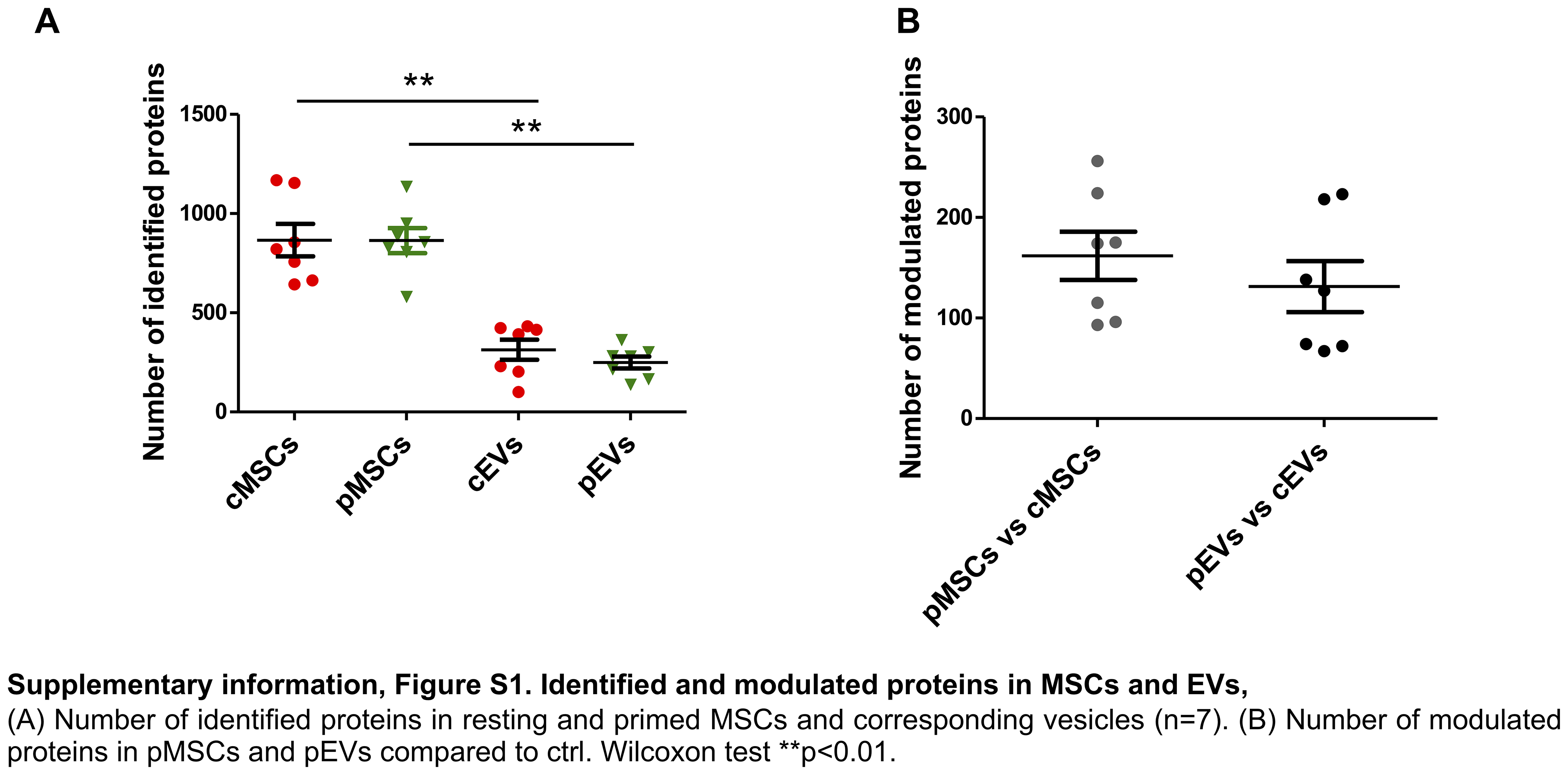

Supplement: Supplementary file 10 [file Image_1.TIFF]

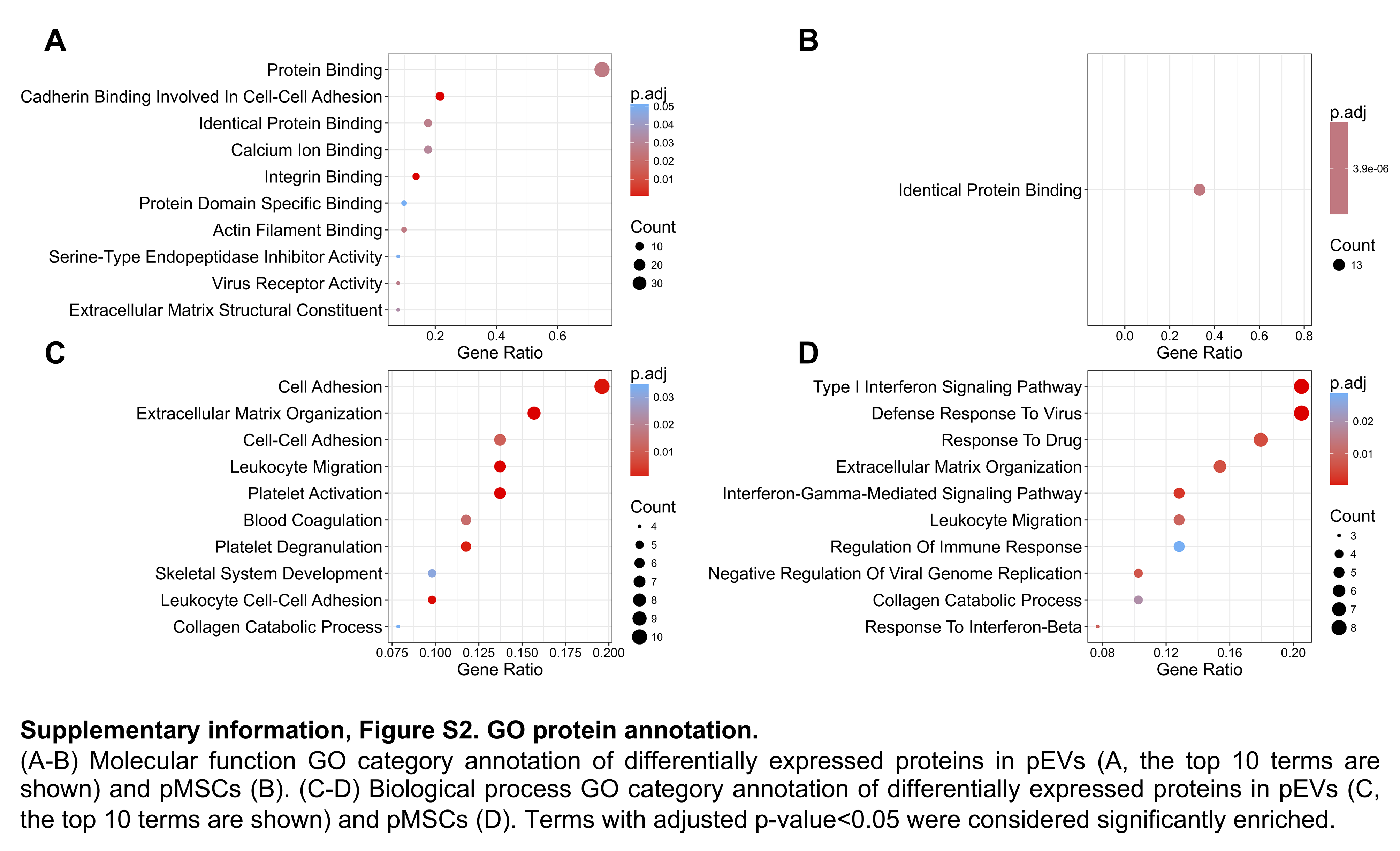

Supplement: Supplementary file 11 [file Image_2.TIFF]

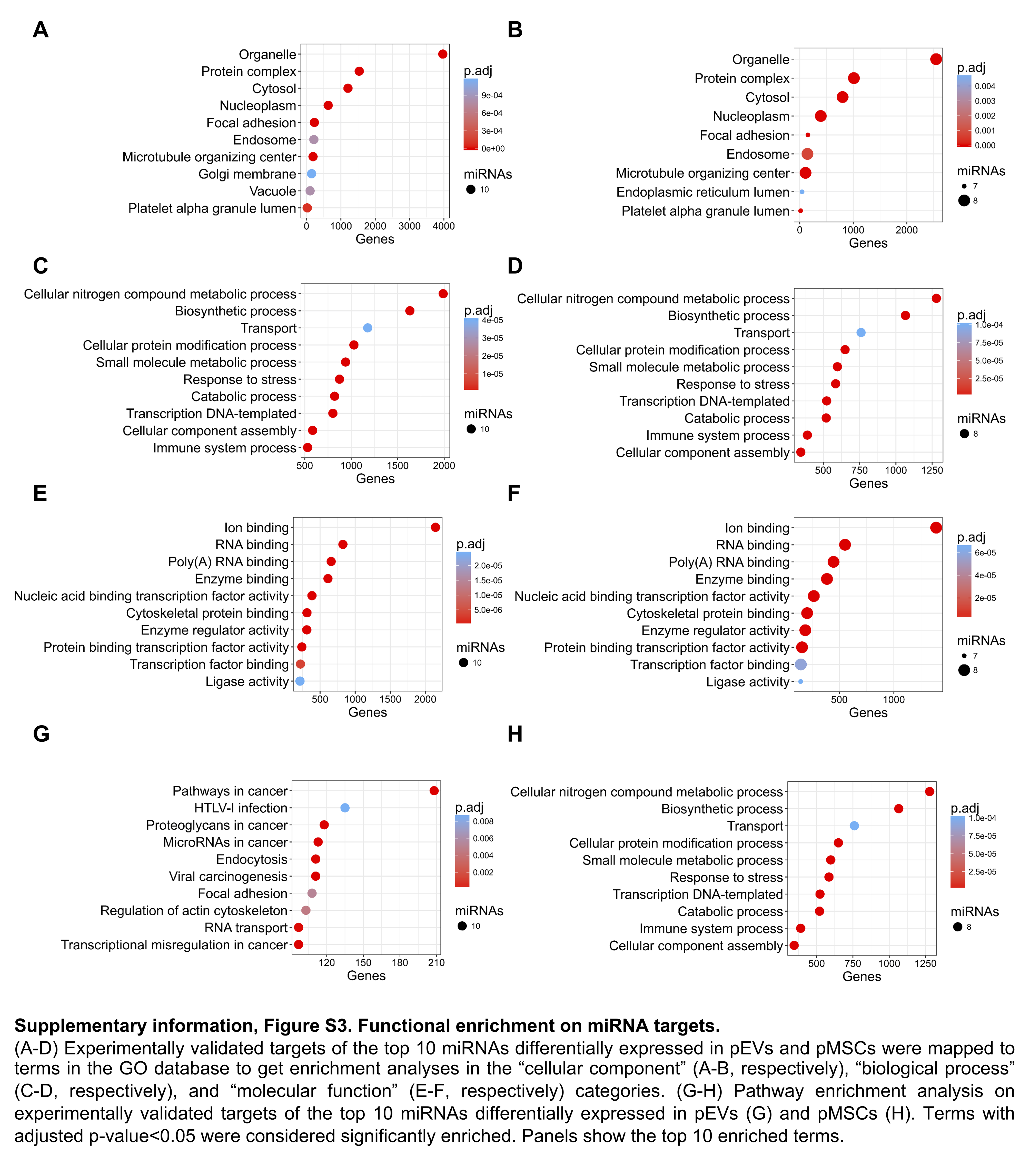

Supplement: Supplementary file 12 [file Image_3.TIFF]
